# A community response approach to mental health and substance abuse crises reduced crime

**DOI:** 10.1126/sciadv.abm2106

**Published:** 2022-06-08

**Authors:** Thomas S. Dee, Jaymes Pyne

**Affiliations:** 1 Graduate School of Education, Stanford University, 520 Galvez Mall, Stanford, CA 94305, USA.; 2 National Bureau of Economic Research, 1050 Massachusetts Ave., Cambridge, MA 02138, USA.

## Abstract

Police officers often serve as first responders to mental health and substance abuse crises. Concerns over the unintended consequences and high costs associated with this approach have motivated emergency response models that augment or completely remove police involvement. However, there is little causal evidence evaluating these programs. This preregistered study presents quasi-experimental evidence on the impact of an innovative “community response” pilot in Denver that directed targeted emergency calls to health care responders instead of the police. We find robust evidence that the program reduced reports of targeted, less serious crimes (e.g., trespassing, public disorder, and resisting arrest) by 34% and had no detectable effect on more serious crimes. The sharp reduction in targeted crimes reflects the fact that health-focused first responders are less likely to report individuals they serve as criminal offenders and the spillover benefits of the program (e.g., reducing crime during hours when the program was not in operation).

## Teaser

Mental health first responders successfully managed targeted police calls, reducing the reporting of and actual crime.

## INTRODUCTION

Police often serve as first responders to emergency calls involving nonviolent individuals in mental health distress or suffering from alcohol or drug abuse. This procedural norm has been the subject of debate and criticism for two broad reasons. One is that serving as first responders to calls involving mental health crises is a substantial drain on scarce police resources and comes with heavy human and social costs, even in the absence of police violence and use of lethal force (*1*–*4*). Police currently spend more time responding to such “low-priority” calls than to any other type of emergency call (*5*). Recent estimates (*6*, *7*) suggest that a quarter to two-thirds of the emergency calls involving disorder, mental health, medical, and noncriminal calls to which police currently respond could instead be directed to mental health crisis experts and other first responders (i.e., a “community response” model). Those charged with minor offenses such as loitering, making false statements, and vandalism cost the criminal justice system roughly $500 to $600 per offense and come with even higher additional social costs (*8*). The potential reallocation of resources away from a police response and toward mental health supports is often a part of current initiatives to “defund the police” (*7*, *9*, *10*).

Second, having armed and uniformed police as first responders to a mental health or substance abuse crisis may increase the likelihood of costly outcomes and inappropriate care. Individuals living with serious mental illness are no more prone to violence or unpredictability than the general population (*11*, *12*). However, having police officers as first responders to a mental health crisis can result in unnecessarily violent and tragic outcomes (*13*, *14*). Recent news coverage (*15*–*17*) has drawn public attention to particularly shocking incidents in which responding police officers seriously harmed or killed a person in mental health distress (*18*). More generally, having the police respond to such incidents can be costly and unproductive because police are more likely than mental health clinicians to direct individuals experiencing a mental health episode to the criminal justice system rather than to the appropriate health care (*13*).

In response to these concerns, municipalities across the country have begun to pilot targeted reforms. The two most common approaches augment the capacity of police officers to serve as effective first responders to individuals experiencing mental health crises. The “crisis intervention team” (CIT) approach emphasizes training police officers how to respond to individuals in crisis and connect them with appropriate services (*19*). In contrast, the “co-response” model involves structuring explicit partnerships between police departments and professional mental health practitioners so they can simultaneously respond to incidents involving mental health crises (*20*–*23*). A third and less common approach either delays or foregoes on-scene police involvement in certain incidents by relying on “a new branch of civilian first responders known as ‘Community Responders’” (*5*). These so-called community response programs can use first responders with expertise in a breadth of social service support and establish a triage protocol under which emergency calls for mental health crises are first addressed by a health team (e.g., a mental health crisis interventionist and a paramedic) before deciding whether to request direct police involvement (*5*).

The momentum behind the adoption of programs that seek to improve police interactions with individuals in mental health crises has motivated multiple empirical studies that seek to understand their impact. Several systematic reviews and meta-analyses have synthesized this evidence, particularly focusing on the more common CIT and co-response models (*20*, *24*–*28*). In general, this empirical literature suggests that these program innovations have beneficial effects by reducing arrests and detention rates, but evidence is mixed on whether these programs are cost-effective. However, the research designs used in these studies (e.g., case notes, qualitative and descriptive studies, before/after comparisons, and cross-sectional comparisons) generally do not support credible causal inference. For example, one recent prominent review concludes that “… we caution against drawing conclusions related to causality based on these findings” (*27*). There is a similar lack of evidence on the impact of less common community response models. Existing evaluations are typically conducted internally by cities, police departments, or community response teams and rely on descriptive evidence of the number of calls taken by the few community response units operating across the United States (*5*).

Furthermore, critics warn that initiatives to reduce police involvement in response to emergency calls will “embolden the bad guys” (*29*) and unintentionally increase the prevalence of more serious criminal offenses (*30*–*33*). This belief, often referred to as the “broken windows” theory in which police response to low-priority criminal violations prevents more serious ones, underscores the need for research studies that can provide credibly causal estimates of the impact of these innovative programs both on the focal, less serious crimes they target and on more serious offenses. However, the debate on defunding police has a limited causal basis, having “proceeded without adequate research about either the scale or nature of issues that the police handle or the potential consequences of the proposed reform efforts” (*7*). This study seeks to provide such evidence by examining the impact of a community response program recently piloted in the City and County of Denver, Colorado through the independent analysis of a preregistered, quasi-experimental design coupled with several complementary robustness checks.

### The Support Team Assistance Response pilot program

The Support Team Assistance Response (STAR) program in Denver provides a mobile crisis response for community members experiencing problems related to mental health, depression, poverty, homelessness, and/or substance abuse issues. The STAR response consists of two health care staff (i.e., a mental health clinician and a paramedic in a specially equipped van) who provide rapid, on-site support to individuals in crisis and direct them to further appropriate care including requesting police involvement, if necessary. The design of the STAR program is based on the Crisis Assistance Helping Out On The Streets program developed in Eugene, Oregon (*34*).

STAR began operations on 1 June 2020 for a designated 6-month pilot period. During this period, STAR limited its operations to selected 911 calls for assistance in eight purposefully chosen police precincts (i.e., out of the city’s 36 precincts), where the need for STAR services was anticipated to be the greatest. The pilot area was in the central downtown area of Denver (fig. S1) and largely represents neighborhoods with residents who are more affluent, educated, and white than the city as a whole (see table S1). However, all but one of the neighborhoods in the STAR pilot service area are also designated by the city as “displacement-vulnerable” areas, rapidly gentrifying city spaces where poor and otherwise at-risk residents are being pushed out (*35*). In such contested urban spaces, there are often increasing demands on police to conduct “rabble management” that addresses overwhelmingly nonviolent incidents (*36*–*39*).

Operators responding to 911 calls for assistance dispatched STAR staff to eligible incidents that were located in the designated police precincts and during the program’s hours of operation (Monday to Friday, 10 a.m. to 6 p.m.). The identification of emergency calls eligible for STAR services relied on two specific screening criteria. First, the incident had to designate at least one of several codes: calls for assistance, intoxication, suicidal series, welfare checks, indecent exposure, trespass of an unwanted person, and syringe disposal (*40*). Second, to dispatch the STAR van, there needed to be no evidence that the incident involved serious criminal activity, such as weapons, threats, or violence, or serious medical needs. The STAR team also responded to calls from uniformed police to engage with community members in crisis and initiated engagement in the field on their own. Over the 6-month pilot period, the STAR team responded to 748 incidents or nearly 6 incidents per 8-hour shift. Roughly a third of calls to STAR occurred at the request of responding police, while the rest were due to a direct 911 dispatch or to the STAR team responding independently to a field observation—none of which required a call to police for assistance or for a response to a criminal offense (*41*).

### Measuring STAR impacts on crime

We identify the impact of the STAR program on STAR-related and STAR-unrelated measured crime using “difference in differences” (DD) and “difference in difference in differences” (DDD) designs that effectively rely on before-after comparisons across treated and comparison precincts (i.e., along with the evidence from several complementary robustness checks and alternative estimation procedures). To identify the impact of the STAR program, we consider all criminal offenses reported by the City and County of Denver through data collected as part of their participation in the federal National Incident-Based Reporting System (NIBRS). These data include calls for police assistance that escalated to offenses reported by the police regardless of whether they led to formal charges (including arrest) or whether the STAR team was dispatched or responded to the call. However, offenses are not differentiated by whether they led to an arrest or some other offense-related outcome (e.g., a citation). We also do not have access to such arrest and citation data and recognize their possible confounding. That is, such data may be missing (e.g., valid criminal offenses where no one is apprehended and, therefore, there is no arrest or citation) and may be confounded further by police and prosecutorial discretion around whether to sustain an arrest or citation for a given offense.

Before our analysis, we coded each offense as directly related to STAR operations (e.g., disorderly conduct, trespassing, alcohol, and drug use) or not (e.g., burglary; see tables S2 and S3 and Supplementary Text for details). For this focal outcome (i.e., lower-level reports of criminal offenses), we expect either the STAR team or the police to often engage the individual in question. If a criminal offense is recorded during such service calls, then it implies either an arrest or a citation. If no crime is recorded, then it implies either a field determination that no criminal offense occurred or a discretionary decision not to record such low-level criminal offenses (e.g., trespassing).

The impact of the STAR program on the frequency of these offenses is theoretically uncertain. For example, to the extent police who respond to mental health and substance abuse incidents consistently direct individuals in crisis to health care services without also identifying them as low-level criminal offenders, the overall effects of the STAR program would be muted—or even null. The Denver police have participated in CIT training designed to support their capacity to identify individuals who need mental health support and to direct those individuals to appropriate care. Because the comparison condition in this study consists of such CIT-trained police as first responders, the introduction of the STAR team could, in theory, have small or nonexistent effects on recorded crime.

This study provides quasi-experimental evidence on the overall (i.e., “reduced form”) effect of STAR’s community response approach on the number of recorded crimes. With respect to reducing recorded criminal offenses, the STAR program’s overall impact could reflect the combination of two broad, underlying mediating mechanisms that merit careful emphasis. One involves program-induced reductions in the recording of existing criminal offenses, while the other concerns reductions in actual crime.

The first mediating mechanism would occur when STAR first responders simply do not record an existing criminal offense (e.g., substance abuse and disorderly conduct) that police officers would record when responding to a given incident. This reporting mechanism has clear empirical relevance given that (i) under NIBRS procedures, law enforcement officials (i.e., not STAR staff) identify and report criminal offenses and (ii) the program data from the period we study indicate that STAR staff did not involve police in their service calls (*41*). However, this reporting mechanism also reflects an impact of social consequence. Specifically, it implies that, when STAR staff replace police as first responders, individuals in mental health or substance abuse crises may be more likely to receive health care and are less likely to be identified as criminal offenders (i.e., implying arrests or citations).

A second class of mediating mechanisms underlying STAR’s overall impact also reflects effects of clear policy relevance. Specifically, there are several reasons that the STAR program could also lead to a genuine reduction in the prevalence of criminal offenses. First, this would occur if the STAR team is more effective than police in implementing de-escalation tactics that reduce the likelihood of further criminal acts (e.g., assaults) when responding to an incident (*42*, *43*). In addition, the STAR team may prevent crime in the near future by reducing recidivism among individuals in crisis. Individuals experiencing mental health or substance abuse crises are quite likely to reoffend (*42*). However, STAR’s targeted provision of health care could reduce the prevalence of such future incidents that would otherwise be recorded as crimes.

In addition, there are at least two other potential “spillover” mechanisms by which the STAR program reduces crime. One is the possibility that the presence of the STAR program in a precinct improves police officers’ implementation of their CIT training. This can occur if officers are more likely to call the STAR team when in need or if they better implement their own CIT training by independently directing individuals in mental health crises to health care responders rather than the criminal justice system when they know STAR is active in their precinct. Another possible, although uncommon, mechanism happens when STAR staff initiate a response in the field. If STAR staff happened to observe an individual clearly in need of their services, then they would sometimes respond without evidence that a crime had yet occurred and without direction from 911 dispatchers or on-scene police officers (*41*).

A reduced-form analysis similar to the one used in this study cannot exactly decompose STAR’s overall impact on crime into the components attributable to these varied mechanisms (e.g., genuine crime reductions and the differential recording of individuals in mental health crises as not having committed criminal offenses). However, we do discuss two pieces of ancillary evidence that indicate whether STAR’s overall impact partly reflects lower levels of actual crime in addition to the reduced reporting of criminal offenses. First, we provide direct evidence for genuine crime reductions by examining the impact of the STAR program on crimes occurring outside STAR’s operating hours (e.g., spillover benefits due to reduced recidivism). Second, we compare the total crime reduction attributable to STAR to the amount that would be expected if STAR’s effects only operated through its service calls. We construct this expected number by multiplying the number of STAR service calls conducted during the pilot period by the number of criminal offenses typically recorded in such criminal incidents during the pretreatment period.

## RESULTS

**Fig. 1.**
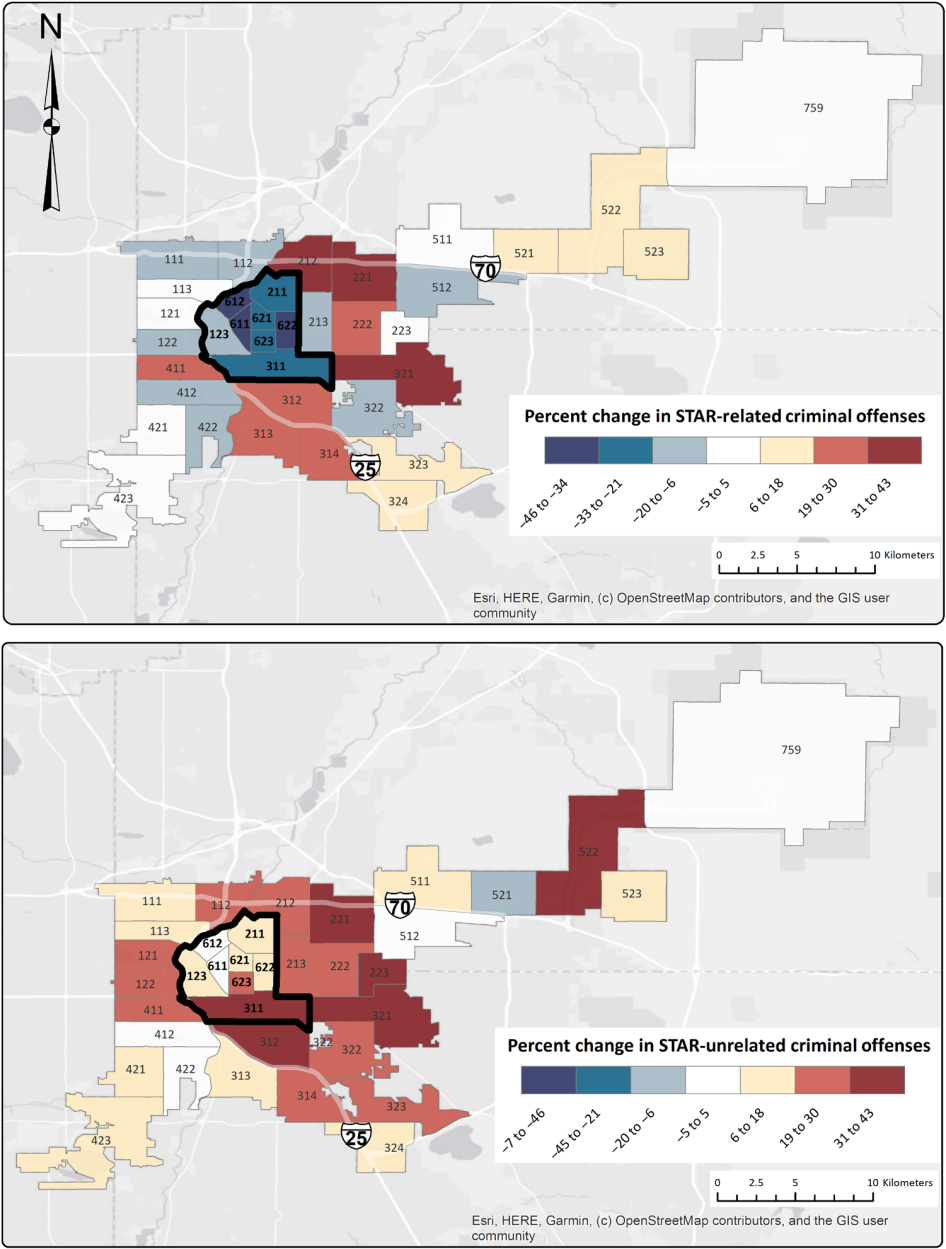
Changes in criminal offenses before and after the STAR pilot implementation. Thick black lines surround the police precincts where the STAR program was active.

As an initial and unrestrictive way to visualize the impact of the STAR program, Fig. 1 shows precinct-level maps that illustrate the before-after changes in measured crime by offense type. These crime measures consist of all incidents reported to the Denver police department that they then record as involving criminal activity, regardless of whether an arrest occurred. Our main analysis focuses on monthly, precinct-level data for the period 6 months before and after the start of the STAR pilot program (i.e., December 2019 to November 2020 in 432 precinct-month observations), differentiating offenses as STAR-related and STAR-unrelated offenses (see Supplementary Text for details). These maps show that treated precincts experienced sharp, comparative declines in STAR-related crimes but not in those crimes not directly related to STAR services.

**Fig. 2.**
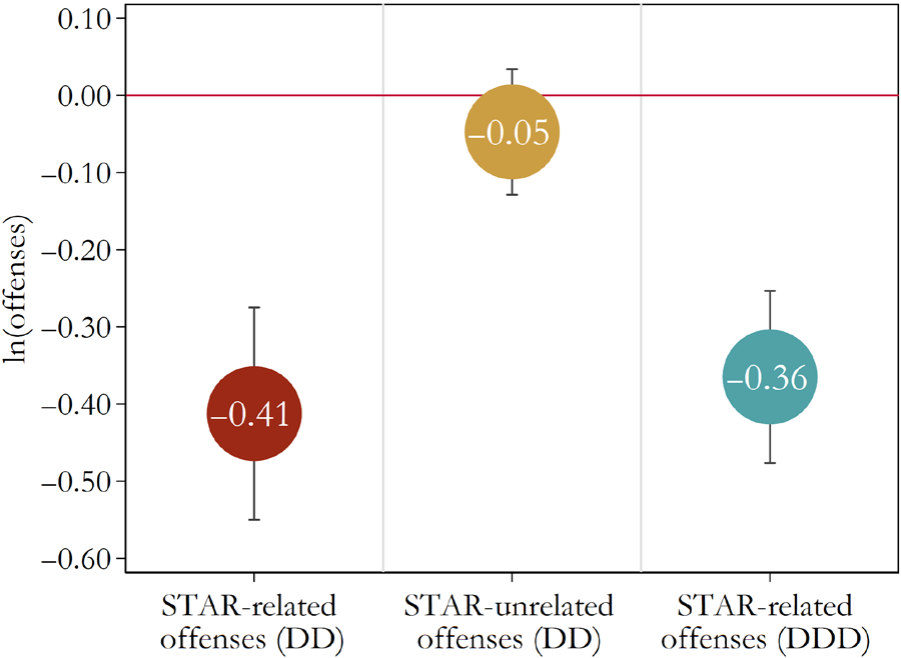
Estimated effects of the STAR program on criminal offenses. The DD estimates are based on 432 precinct-month observations and condition on precinct fixed effects and month fixed effects. The DDD estimates are based on the stacked precinct-month data for STAR and non-STAR offenses (*n* = 864). The DDD estimates condition on fixed effects unique to each category of the following two-way interactions: precinct-by-month offense, precinct-by-STAR offense, and month-by-STAR offense. The outcome variables are the natural log of the offense counts. Dots are the coefficients; bars are 95% confidence intervals. See table S4 for numerical results.

Estimates based on the DD and DDD designs (i.e., Eqs. 1 and 2) allow us to estimate these effects directly and to condition on fixed effects unique to precincts and months. Figure 2 displays the key estimates (and corresponding 95% confidence intervals) based on these quasi-experimental specifications. For our main confirmatory hypothesis, the DD estimate indicates that the STAR program led to large and statistically significant reduction in the targeted measured crimes (*b* = −0.41, SE = 0.07, *t* = −6.09, *P* < 0.001). This estimated impact on the natural log of STAR-related crimes implies that the program reduced these targeted crimes by 34% [i.e., (*e*^(−0.41)^ − 1) × 100]. By contrast, the estimated effect of the STAR program on measured crimes that were not directly related to STAR services was comparatively small and statistically insignificant (*b* = −0.05, SE = 0.04, *t* = −1.18, *P* = 0.245). This finding suggests that the targeted fielding of mental health professionals as first responders did not increase the frequency in reporting more serious criminal incidents in treated precincts. This null result can also be understood as affirming the causal warrant of the DD design by indicating that there were not unobserved and confounding determinants of crime unique to the precincts and months associated with the STAR pilot, a finding consistent with the causal warrant of the DD design. The DDD specification (i.e., Eq. 2) leverages these comparative results by using the data on crimes unrelated to STAR operations as a comparison condition unique to each precinct-month observation. The DDD estimate similarly implies that STAR operations led to a large and statistically significant reduction in reports of targeted crimes (*b* = −0.36, SE = 0.05, *t* = −6.64, *P* < 0.001). This DDD estimate suggests that the STAR program led to a 30% reduction [i.e., (*e*^(−0.36)^ − 1) × 100] in STAR-related offenses (see table S4 for full numeric results).

**Fig. 3.**
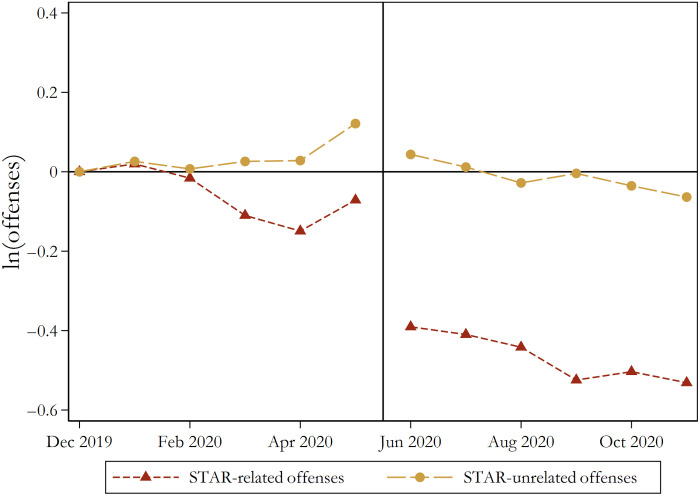
Event-study model. The DD event-study estimates are based on 432 precinct-month observations and condition on precinct fixed effects and month fixed effects. The outcome variables are the natural log of the STAR-related and STAR-unrelated offense counts. The event-study estimates identify for each outcome the regression-adjusted treatment comparison differences by month relative to the first time period (i.e., December 2019). The vertical line separates pretreatment months from the months after the STAR pilot program that began in June 2020. See table S5 for numerical results.

Figure 3 illustrates the key estimates from event-study DD specifications that allow for effects unique to treated precincts in each month before and after the onset of STAR operations (see Supplementary Text for specification details). The bottom red line in Fig. 3 represents the point estimates from an unrestrictive event-study specification that examines the treatment comparison difference in reports of STAR-targeted crimes in the months before and after program implementation (see table S5 for numeric results). The top yellow line presents similar estimates based on STAR-unrelated offenses. These results indicate that, in the months before STAR operations, the treated and comparison precincts had similar trends in STAR-related crimes. More formally, we do not reject the hypothesis that the effects on STAR-related measured crimes that are unique to treated precincts in the months before STAR operations are the same as those in the comparison precincts (*P* = 0.71). These results are consistent with the “parallel trends” assumption of the DD design and with a causal interpretation of the results based on Eq. 1. The event-study estimates in Fig. 3 also illustrate the distinct drop in STAR-related crimes associated with the onset of STAR services, as well as the comparative absence of any relationship with the prevalence of crimes that are not directly related to STAR operations. Figure S2 similarly presents conditional means for each group of offenses (i.e., STAR-related and STAR-unrelated) by month for both treatment and comparison precincts, and fig. S3 shows trends in STAR-related offenses among the eight treated districts.

The Supplementary Materials presents several ancillary analyses that explore the robustness of these findings. For example, Poisson and negative binomial specifications that recognize both the count nature of the crime data and the presence of fixed effects (*44*) return results similar to those based on ordinary least squares estimates of Eq. 1 (table S6). The DD results presented here are also similar in specifications that rely only on when treating May 2020 as a treatment month among STAR-active precincts to allow for anticipation effects (table S6). When we remove offenses that are STAR related but not STAR targeted (i.e., simple assault, simple assault on a police officer, and disarming a police officer; see Supplementary Text for details), the static DD effect size is larger than what we report. That is, the point estimate is −0.41 when those assault offenses are included as STAR-related offenses and is −0.49 when they are not (table S6; see Supplementary Text for additional details). We also find similar levels of statistical significance when we remove police precinct 311, which is not entirely serviced by the program, and in specifications that correct for the potential finite-sample bias in the precinct-specific clustering of the error term (table S6). We also find that the results are robust when using permutation-based randomization inference (fig. S4).

Next, to test whether common seasonal changes in crime rates threaten the causal interpretation of these results, we construct parallel “placebo effect” datasets of months from December 2016 to November 2017, December 2017 to November 2018, and December 2018 to November 2019. In each time frame, we code all months in each dataset past May as a placebo “treatment” month for all STAR-active precincts. If these estimates indicate a consistent drop in measured crime in the STAR precincts following May of each year, then that would suggest that the main results reflect seasonal patterns rather than the implementation of the STAR pilot. Instead, results based on these data consistently suggest no statistically detectable reductions in the measured crime after June in any of these prior years. We show these prior-year placebo estimates for static DD specifications (table S6) and event studies (figs. S5 to S7).

Several additional internal validity checks detailed in the Supplementary Material provide evidence on the coronavirus disease 2019 (COVID-19) pandemic as a possible confound and related evidence that speaks more generally to the validity of the parallel-trends assumption. In particular, estimates near the bottom of table S6 indicate that the estimated effects of the STAR program are similar in specifications that only rely on data following the beginning of COVID-19 shutdown orders (i.e., March 2020 to November 2020). Second, the estimated effects of the STAR program are also similar when based on generalized synthetic control design (*45*) and comparative interrupted time series design (*46*) that explicitly accommodate the presence of preexisting trends across treatment and comparison precincts (see table S7 for results and Supplementary Text for details on these procedures).

Last, the Supplementary Materials also presents analyses that explore the potential heterogeneity in these results. For example, the event-study results (Fig. 3) suggest that the impact of the STAR program grew over time. However, estimates based on a semi-dynamic DD specification cannot reject the hypothesis that the impact of the STAR program is the same in each of the 6 months of operation (*P* = 0.91; table S5). In addition, in table S6, we show that DD specifications that allow for spatial spillover effects of the STAR program in geographically adjacent districts indicate that the estimated effect of STAR operations on neighboring precincts was small and statistically insignificant. However, there do appear to be meaningful temporal spillover benefits of the STAR program within treated precincts. Specifically, the reductions in STAR-related measured crimes in treated precincts also occurred during days of the week and times when the program was not active (table S6). This pattern is consistent with the hypothesis that the STAR program provided helpful services to individuals in crises that were somewhat persistent rather than brief and episodic. However, these temporal spillovers do not extend to precincts adjacent to those with STAR operations.

There remains the possibility of spatiotemporal spillover. For example, “near repeat” crime is the phenomenon in which criminal incidents increase near to and shortly after the occurrence of a crime (*47*). However, our analysis relies on precinct-level panel data (i.e., the level at which STAR was implemented), while spatiotemporal spillovers are likely to occur on finer spatial levels. Even so, we estimated effects of the STAR program on STAR-adjacent precincts during STAR-active and STAR-inactive time periods. The results are consistently null (see table S6). While these findings indicate that there are no cross-precinct spillovers, we note that this is not necessarily evidence against the near-repeat phenomenon. Furthermore, using the police categorization of crimes suggests that STAR operations led to a 14% reduction in overall crimes (table S8) and that these reductions were concentrated in three STAR-related categories (i.e., alcohol and drugs, disorderly conduct, and other crimes against persons).

## DISCUSSION

Police officers in the United States currently spend a substantial amount of their time responding to nonviolent emergency calls for assistance, which often involve individuals experiencing mental health or substance abuse crises. However, police officers are not extensively trained to assist with such crises and most believe that such incidents are outside of their professional purview (*48*, *49*). As a result, emergency calls for assistance may be engaged as criminal violations, sometimes with unnecessarily violent or even tragic consequences, when they can be better addressed as health issues. The widespread recognition of this issue has motivated initiatives to improve police training and cooperation with health professionals (e.g., CITs and co-response models). A less common but more marked innovation for responding to nonviolent individuals in crisis is to delay or forego police involvement by sending a health care team as first responders (i.e., a community response model). Although each of these programmatic models is grounded in a sensible theory of change, there is not currently credible, causal evidence on their effects (*5*, *7*, *20*, *24*–*26*, *28*).

In this study, we have presented the results of a preregistered quasi-experimental design that examined the effects on crime of a community response program that dispatched a mental health clinician and paramedic to nonviolent emergency calls rather than first sending police. STAR is a community response program that operated as a pilot program for 6 months and provided service within eight police precincts in Denver’s central downtown area. Drawing on data of calls for service that escalate to criminal offenses recorded by police officers (i.e., incidents leading an arrest or citation), from December 2019 to November 2020, we have used a DD model that effectively compares the changes in police-recorded criminal offenses both before and after the pilot program and across the treated and untreated precincts. We complement the results of this preregistered design with a variety of robustness checks (e.g., alternative approaches to estimation and inference and falsification exercises based on prior years of data). We also examined whether STAR operations influenced the frequency of more serious offenses that were not directly targeted by the program and found no discernable impact.

We find that the program led to large and sustained reductions in reports of STAR-related offenses in treated precincts, while unrelated offenses over the treatment period changed little in those same police precincts (Fig. 2). Our comparative estimates suggest that the service reduced the number of STAR-related offenses in treated precincts by 34% over the 6 months of the pilot phase. While the average number of STAR-related offenses in our precinct-month sample is 34 (see table S3), the frequency of these measured offenses in STAR-active precincts before treatment is much higher (i.e., averaging 84.3 offenses per precinct-month from December 2019 to May 2020). This impact estimate implies that the STAR pilot program prevented nearly 1400 criminal offenses within the eight participating precincts and the 6 months of operation (i.e., 84.3 × 0.34 × 8 × 6 = 1376). This program-induced reduction in measured offenses is broadly consistent with the scale of STAR operations. Specifically, the STAR team responded to 748 calls during our study window. At baseline (i.e., during the pretreatment period), each STAR-related incident resulted in an average of 1.4 recorded offenses in treated precincts. This suggests that we should expect 748 field calls by STAR staff to result directly in just over 1000 fewer recorded offenses (i.e., 748 × 1.4 = 1047).

The overall (i.e., reduced form) estimated impact of the STAR program (i.e., 1376 fewer criminal offenses) reflects the simultaneous influence of two distinct, broad, and policy-relevant mechanisms. One is due to STAR first responders providing health care to individuals in mental health or substance abuse crises simply not recording them as low-level criminal offenders subject to arrest or citation. The second mechanism is due to actual reductions in crime. The empirical relevance of these two complementary mechanisms cannot be exactly identified. However, the fact that the total reduction in criminal offenses attributable to STAR (i.e., 1376) clearly exceeds the highest number of criminal offenses likely to have been confronted by STAR staff (i.e., 1047) suggests that the STAR program reduced actual crimes. In addition, the evidence that the STAR program reduced the number of low-level criminal offenses during hours when the program was inactive (e.g., reducing recidivism among individuals in crisis) provides direct support for the existence of this underlying mechanism.

Last, we find that STAR’s operation during the pilot phase did not increase reports of more serious or violent offenses. Under the broken windows theory, less police enforcement of low-priority criminal violations will increase the prevalence of more serious and violent criminal offenses being recorded (*32*). Our evidence suggests that this was not the case in Denver’s treated precincts. The absence of such an impact also provides an indirect validation of our main finding by indicating that there were not confounding trends in crime unique to treated precincts. The DDD estimates we present formalize this robustness check but rely on additional assumptions (e.g., there is no crime-fighting benefit to having STAR in a precinct). Our findings’ interpretation as robustness checks is thus only secondary to the standalone finding of null effects on serious crimes as a test of prior criminological theory.

The evidence in this study indicates that the STAR community response program was effective in reducing police-reported criminal offenses (i.e., both reducing the designation of individuals in crisis as criminal offenders and reducing the actual level of crime). These results provide a compelling motivation for the continued implementation and assessment of this approach. However, successfully replicating the STAR program is likely to rely on key implementation details such as the recruitment and training of dispatchers and mental health field staff as well as the successful coordination of their activities with the police. Furthermore, the generalizability of the community response approach to a broader set of potentially preventable charges is uncertain and a design feature worthy of further study. There are also additional details about programs such as STAR that merit further investigation and clarification. For example, we are unsure of whether the existence of STAR may have increased the trust and the willingness of community members to call 911. However, we note that such an effect is likely to imply that our estimates underestimate the true effect of the STAR program. That is because increase in trust and willingness to call 911 is likely to increase measured crime in the short run as some of these calls would result in police engagement regardless of arrest status. Future studies may also consider the effects of programs like STAR on health-related outcomes, such as access to health services (e.g., counseling and therapy) and related measures of well-being.

Another important policy consideration is its cost-effectiveness. The total cost of the 6-month STAR pilot program was $208,141 (*50*). One useful way to frame this public outlay is to note that the corresponding reduction of 1376 offenses implies a cost of $151 per offense reduced. To put this in perspective, the available estimates (*8*) suggest that the direct criminal justice cost for a minor criminal offense (e.g., imprisonment and prosecuting) averages $646 (in 2021 dollars). In other words, the direct costs of having police as the first responders to individuals in mental health and substance abuse crises are over four times as large as those associated with a community response model. A fuller reckoning of the costs and benefits associated with community response models would also include the costs and benefits associated with any health care brokered by the first responders. For example, police officers may be more likely than community responders to direct individuals in crisis to comparatively expensive emergency room care or to no care at all. Nonetheless, the results presented here suggest that community response models merit careful consideration as a highly cost-effective way to reduce police engagement with nonviolent individuals in crisis and to instead respond with appropriate health care.

## MATERIALS AND METHODS

We collected counts of measured criminal offenses using data made publicly available by the City and County of Denver, Colorado through their Open Data Catalog (ODC) and based on the NIBRS. Because Denver’s reporting is NIBRS-compliant, a mere civilian call is not, by itself, sufficient to imply that a criminal offense is registered. Specifically, the NIBRS User Manual notes that “Participation in NIBRS requires law enforcement agencies to report certain facts about each criminal incident coming to their attention within their jurisdictions.” However, because at least some calls will involve a complaint without clear criminality, they will not rise to the level of a “criminal incident coming to their attention.” Thus, not every complaint or incident investigated by police will result in an offense being recorded in our data—instead, only those incidents that result in an arrest or citation are recorded as offenses.

Our coding identifies the types of reported offenses the STAR program would conceptually be expected to reduce (i.e., “STAR-related” offenses) rather than just the kinds of incidents the program targets. “Disarming a peace officer” is an illustrative example of the distinction between STAR-related and STAR-targeted offenses. The dispatch protocol would not send out a STAR team in response to such an offense (i.e., it is not STAR-targeted). However, this is exactly the sort of offense that might be reduced by having mental health specialists as first responders rather than police (i.e., it is STAR-related).

However, there are differences between the data reported to NIBRS and what is reported on Denver’s ODC that we use here. That is, NIBRS records all serious incidents such as homicide and arson but only reports records of arrests made for less serious offenses such as the STAR-related ones that are our focus here. Conversely, we have confirmed through correspondence with M. Lunn, the Manager of Strategic Initiatives for the Denver Police Department, that their data record not only those crimes resulting in arrests or formal charges (i.e., ones involving subsequent prosecutorial decisions) but also all criminal incidents recorded by the police. To that end, our main results identify program-induced reductions in substantiated criminal incidents identified by or reported to the police. Conceptually, these reductions combine the relabeling of existing behaviors that occur when individuals in crisis receive health care rather than being directed into the criminal justice system and a reduction in criminal offenses by individuals in crisis who would offend repeatedly in the absence of health care.

The ODC contains incident-level data on all criminal offenses reported to law enforcement from January 2016 to November 2020. From that data catalog, we constructed a panel dataset of criminal offenses observed in each of 36 precincts over each of 12 months for the period from December 2019 to November 2020 (i.e., 432 precinct-month observations). This period includes the 6-month pilot phase and the 6 months before the pilot phase.

The single confirmatory hypothesis in our preregistered analysis plan (https://osf.io/nqhvf) focuses on the impact of the STAR program in a static DD specification that takes the following form

$$Y_{\mathrm{pm}}=\alpha_{p}+\gamma_{m}+{\theta S}_{\mathrm{pm}}+\varepsilon_{\mathrm{pm}}$$ (1)

where *Y_pm_* is the natural log of STAR-related criminal offenses for precinct *p* in month *m*. The term, *S_pm_*, is a binary indicator equal to 1 only for STAR-participating precincts observed during the period when the program was active. The coefficient of interest, θ, represents the effect of the STAR program conditional on fixed effects unique to each precinct and to each month (i.e., α*_p_* and γ*_m_*, respectively). The term, ε*_pm_*, is a mean-zero error term with clustering at the precinct level. The static DD specification in Eq. 1 embeds the assumption that the STAR program implies a one-time level shift in crimes. To explore possibly time-varying treatment effects, we also report the results of “semi-dynamic” DD that unrestrictively allow for effects to vary uniquely in each of the six treatment months. In the Supplementary Materials, we also present the results based on versions of Eq. 1 that use alternative approaches to estimation (e.g., Poisson and negative binomial count data specifications) and to inference (e.g., adjustments for finite sample clustering bias and randomization inference).

This DD research design effectively compares the before/after level of measured crimes in STAR-active precincts to the contemporaneous change in comparison precincts (i.e., those where STAR services were unavailable). A key identifying assumption of this design is that the time-varying changes within the comparison precincts provide a valid counterfactual for what would have happened in the treated districts in the absence of treatment. We examine the empirical validity of this assumption in two ways. One is to estimate “event study” DD specifications that unrestrictively allow for effects unique to treatment precincts in each month. The event-study estimates indicate the extent to which the treatment and comparison precincts had similar month-to-month variation in STAR-related crimes before the pilot began (see the Supplementary Materials for details). If treatment and comparison precincts have similar trends in STAR-related crimes in the months before STAR operations, then it would be consistent with the internal validity of the DD design.

A second, important robustness check is to use Eq. 1 to estimate the impact of STAR operations on reports of more serious criminal offenses that are not directly related to STAR operations. If the estimates based on Eq. 1 are reliable, then we would expect the DD design to indicate that the effect of STAR operations on STAR-unrelated crimes is comparatively small, if not indistinguishable from zero. However, if DD estimates indicate that STAR operations had large effects on measured crimes unrelated to STAR, then it would suggest the existences of unobserved and confounding variables that are unique to the treated precincts in the treatment period. We formalize the idea of using the measured crimes unrelated to STAR operations as a comparison condition that is unique to each precinct and month in DDD specifications. Specifically, we stack the precinct-by-month data for these two crime categories (*n* = 864) and estimate the following specification

$$Y_{\mathrm{pom}}=\alpha_{\mathrm{pm}}+\gamma_{\mathrm{mo}}+\delta_{\mathrm{po}}+{\theta S}_{\mathrm{pom}}+\varepsilon_{\mathrm{pom}}$$ (2)

This specification includes unrestrictive fixed effects unique to each possible two-way interaction: precinct-month (α*_pm_*), month-offense (γ*_mo_*), and precinct-offense (δ*_po_*). Critically, the DDD specification controls for unobserved determinants of crime unique to each precinct-month combination. The parameter of interest reflects the estimated effect associated with the three-way interaction unique to STAR-related offenses observed in treated precincts during the treatment period (i.e., *S_pom_*).

## Supplementary Materials

### This PDF file includes:

Supplementary Materials and Methods Supplementary Text Figs. S1 to S7 Tables S1 to S8 References

## Notes

View/request a protocol for this paper from *Bio-protocol*.
