# Supplementary material for: A community response approach to mental health and substance abuse crises reduced crime
Source: Sci Adv. 2022 Jun 8;8(23):eabm2106. doi: 10.1126/sciadv.abm2106 (PMC9176742; doi:10.1126/sciadv.abm2106)
Supplement: Supplementary file 1 — Supplementary Materials and Methods Supplementary Text Figs. S1 to S7 Tables S1 to S8 References [file sciadv.abm2106_sm.pdf]

Science Advances
AAAS

Supplementary Materials for

A community response approach to mental health and substance abuse crises reduced crime

Thomas S. Dee and Jaymes Pyne

Corresponding author: Thomas S. Dee, tdee@stanford.edu; Jaymes Pyne, pyne@stanford.edu

Sci. Adv. **8**, eabm2106 (2022)
DOI: 10.1126/sciadv.abm2106

This PDF file includes:

Supplementary Materials and Methods
Supplementary Text
Figs. S1 to S7
Tables S1 to S8
References

Materials and Methods

City and Police Precinct Characteristics

Figure S1 maps the police districts and neighborhoods in which the STAR program was active. The STAR pilot program operated in select police precincts, "from York St. to I-25 east to west and 38th St. / 40th Ave. to 6th Avenue north to south) and along the South Broadway corridor to Mississippi Ave., with service also being provided to the temporary shelters at the Denver Coliseum and National Western Complex" (*25*). This constitutes mainly the central downtown Denver area and includes police precincts 123, 211, 311, 611, 612, 621, 622, and 623 (see Figure S1). Table S1 presents descriptive demographic and socioeconomic data on these neighborhoods as well as for the entire city based on data from the American Community Survey (ACS). Denver, CO is a city with a population of 678,467. In 2017, the median household income was $59,179 (15% poverty). Just under half (46%) had a bachelor's degree or higher, and the same proportion (46%) are people of color.

Sample Traits

Table S2 shows the categorization of specific offenses in the City's NIBRS-based data as STAR-related or unrelated. Table S3 presents key descriptive statistics based on the precinct-month analytical sample (n = 432). Specifically, this table shows the means for STAR-related and unrelated offenses (i.e., 33.7 and 156.0, respectively). It also shows the offenses across 10 broad and mutually exclusive categories of offenses used in the City's data file. Five uncommon categories of offenses unrelated to the STAR program's mission (i.e., arson, murder, robbery, sexual assault, white-collar offenses) are excluded.

Offense Coding

The incident data identify 199 types of offenses organized into 15 broader and mutually exclusive categories. For our primary analyses, we categorized each recorded offense by whether it was directly related to STAR services. Specifically, prior to our pre-registered analysis, two independent coders rated the categorization of each offense type. Raters had 91 percent agreement on offense type codes (kappa = 0.73). Coders met and reconciled remaining discrepancies. The offenses identified as STAR-related include trespassing, disturbing the peace, possession of illegal drugs, indecent exposure, alcohol violations, loitering, failure to obey police orders, police interference, and public disorder. Prior to STAR operations, the offenses identified as STAR-relevant offenses constituted 20 percent of the offenses reported by Denver police. As a complement to this binary categorization of offenses, we also show results based on broad, mutually exclusive categories the City reports.

Treatment Heterogeneity and Evidence of Robustness

The pre-registered "static" DD specification represented in equation [1] (see main text) assumes that the treatment effect is constant over time. However, the effects of the STAR program could instead have dynamic features. To test for time-varying treatment effects, we also employ a semi-dynamic DD model that unrestrictively allows for treatment effects unique to the month immediately after a precinct first participates and up to five months later:

$$Y_{pm} = \alpha_p + \gamma_m + \sum_{n=0}^{5} \delta_{-n} S_{p,m-n} + \varepsilon_{pm}$$

In this model, the three coefficients of interest are represented by δ_n, which identify the effects of STAR in the first month of the program (i.e., $S_{p,m-0}$) as well as the current effect of having begun

one month earlier (i.e., $S_{p,m-1}$), two months earlier (i.e., $S_{p,tm2}$), and so on. We then test the equivalence of these coefficients of interest using the null hypothesis of a constant treatment effect:

$$H_0: \delta_0 = \delta_{-1} = \delta_{-2} = \delta_{-3} = \delta_{-4} = \delta_{-5}$$

We report the semi-dynamic results, both for DD and DDD specifications, in Table S4. Hypothesis tests consistently fail to reject the null hypothesis of a common treatment effect across the 6-month pilot period.

The main text underscores two types of evidence consistent with the internal validity of the pre-registered DD results. One is the absence of a meaningful impact on offenses rated as unrelated to STAR prior to the analysis (i.e., column 3 in Table S4). Table S5 presents the results of a second and important type of evidence. A central and maintained identifying assumption of our pre-registered DD approach is that the month-to-month outcome changes among comparison precincts (i.e., those without a change in treatment status) provide a valid counterfactual for what would have changed for treatment precincts in the absence of treatment. This "parallel trends" assumption is fundamentally untestable. However, we can provide empirical evidence on the validity of this important assumption through unrestrictive "event study" specifications that allow us to examine whether treatment and comparison group precincts had similar month-to-month changes in outcomes *prior* to the onset of treatment. To the extent that this hypothesis is true, it is consistent with the parallel-trends assumption. We examine this question through event-study specifications of the following form:

$$Y_{pm} = \alpha_p + \gamma_m + \sum_{\tau=1}^{5} \delta_\tau S_{p,m+\tau} + \sum_{n=0}^{5} \delta_{-n} S_{p,m-n} + \varepsilon_{pm}$$

This event-study specification effectively extends the semi-dynamic specification (equation [2]) to allow for fixed effects unique to each month prior to participating in STAR (i.e., "leads" of treatment adoption). That means the coefficients of interest are represented as δ_{-n} and δ_τ, which designate the "effect" for precinct *p* in month *m* of participation in STAR *n* months in the future or τ months in the past. The reference category includes those never participating in STAR and those in six months prior to their first participation in STAR. To examine the assumption of parallel trends, we test whether, *prior* to their participation in STAR, treatment precincts have month-to-month changes in outcomes distinct from comparison precincts:

$$H_0: \delta_5 = \delta_4 = \delta_3 = \delta_2 = \delta_1 = 0$$

We report the event-study results, both for STAR-related and unrelated offenses, in Table S5 and Figure 3 in the main text. The results are consistent with the parallel-trends assumption, indicating that we cannot reject the null hypothesis that the treated precincts had month-to-month changes similar to the comparison districts in the months *prior* to the program activity.

Table S6 presents the key results from a variety of alternative specifications that probe the robustness and heterogeneity of the confirmatory finding. First, we consider alternative approaches to conducting inference in this application. Our main estimates allow for precinct-specific clustering in the error term associated with criminal offenses that is heteroscedastic-consistent. However, because there are only 36 unique precincts, this clustering approach may be subject to finite-sample biases. To examine this concern, we report the results based on the procedure recently introduced by Pustejovsky and Tipton (*51*). The results are quite similar to our reported findings.

As a further and unrestrictive check on our main inference, we also conducted randomization inference with respect to the confirmatory finding. Specifically, over 100,000 replications, we randomly assigned treatment status within precincts and estimated the “impact” of the STAR program. Randomization inference has a particular appeal in applications like this because the data may be better understood as having “design-based” variation in what units are treated rather than having variation due to being drawn from a larger hypothesized population. Figure S4 shows the histogram of estimated effects based on this permutation procedure. Because treatment status was assigned randomly, this distribution can be understood as the distribution of treatment effects when the null hypothesis of no effect is true. Over the 100,000 replications, none of the estimates in this distribution was as large in absolute value as the estimate based on the actual data (i.e., -0.41). This implies a randomization-inference *p*-value that is less than 0.00001.

Table S6 also presents results based on alternative estimation procedures and constructions of the analytical sample. Specifically, Table S6 presents the conditional maximum likelihood (CML) estimates of Poisson and negative binomial specifications that explicitly recognize both the count nature of the offense data and the presence of fixed effects (*28*). The resulting estimates are quite similar to those based on the pre-registered DD specification. Table S6 also presents the main DD results when dropping data from a STAR-participating police precinct (i.e., precinct 311) where program activities were targeted to a main corridor rather than intending to be active precinct-wide. Though there is no clear reason to expect biases from the onset of the COVID-19 pandemic, especially conditional on month fixed effects, Table S6 also shows the results of using data only from March 2020 (i.e., the onset of the shutdown) onward. Both data edits result in DD estimates consistent with our main finding.

Finally, Table S6 also presents the results of exploring two particular forms of treatment heterogeneity. First, we explored the possibility that the STAR program also led to crime reductions in geographically adjacent precincts. Specifically, we created an additional treatment indicator equal to one only for precincts that were adjacent to STAR precincts when the STAR program was active. The estimated effect reported in Table S6 indicates that we cannot reject the null hypothesis of no effect in neighboring precincts. We also explore possibly heterogeneous treatment effects across days of the week and times of the day when the STAR program was active. As the main text notes, the program was only active Monday through Friday, 10AM to 6PM. We created separate counts for STAR-related offenses that occurred within and outside these weekly windows. The results in Table S6 indicate that the STAR program led to similar reductions in targeted offenses across both time periods. As noted in the main text, this finding is consistent with the hypothesis that the program brought into the health-care system individuals in crisis who would otherwise commit police-reported offenses at other times of the week (i.e., evenings and weekends) as well. Table S7 reports results of DD estimates using generalized synthetic control (*45*) and comparative interrupted time series (CITS) designs (*47*), both of which are consistent with our main confirmatory findings.

Table S8 reports DD estimates of the impact of the STAR program on overall offenses and on offenses across the broad and mutually exclusive categories defined in the City’s NIBRS-based data. The point estimates indicate that the STAR program reduced the natural log of total offenses by a statistically significant 0.15, which implies the 14 percent reduction noted in the main text [i.e., $(e^{-0.15} - 1) \times 100$)]. The estimates by category indicate that these reductions were plausibly concentrated in offenses such as “alcohol and drugs” (i.e., -0.53), “disorderly conduct” (i.e., -0.20), and “other crimes against people” (i.e., -0.14).

Pre-Registration Plan

The following is our detailed pre-registration plan, filed on February 14, 2021 prior to any data analysis related to the study.

A. Study Information

1. *Hypotheses*

Precincts participating in the STAR program will have reduced prevalence of criminal offenses related to mental health, poverty, homelessness, and substance abuse in the City of Denver.

B. Design Plan

1. Study type

Observational Study - Data is collected from study subjects that are not randomly assigned to a treatment. This includes surveys, "natural experiments," and regression discontinuity designs.

2. *Blinding*

No blinding is involved in this study.

3. *Study design*

We focus on recorded offenses in each city precinct in a given month from December 1, 2019 though November 30, 2020. This time period represents the six-month pilot phase of STAR (June 2020-November 2020) and the six months prior to the pilot beginning. This design strategy allows us to take advantage of our panel dataset in months surrounding implementation. Our analytical sample consists of 36 precincts and 432 precinct-month observations, from December 2019 through November 2020.

C. Sampling Plan

1. *Existing Data*

Registration prior to analysis of the data

2. *Explanation of existing data*

The data come from open access police records provided by the city of Denver, CO (https://www.denvergov.org/opendata/dataset/city-and-county-of-denver-crime). These data include criminal incident records from January 2, 2016 through January 15, 2021 involving adults. Due to legal restrictions, these data do not report crimes that by nature involve juveniles as victims (e.g., child abuse offenses), suspects or witnesses. These data

also exclude “unfounded” incidents, which authorities have determined did not actually occur after they are reported.

3. *Data collection procedures*

We downloaded open access police records provided by the city of Denver, CO (https://www.denvergov.org/opendata/dataset/city-and-county-of-denver-crime). We retain recorded offenses in each city precinct in a given month from December 1, 2019 though November 30, 2020. This time period represents the six-month pilot phase of STAR (June 2020-November 2020) and the six months prior to the pilot beginning.

4. *Sample Size*

Our analytical sample consists of 36 precincts and 432 precinct-month observations, from December 2019 through November 2020.

5. *Sample size rationale*

This sampling allows for observation of criminal offenses in the city six months before and six months after the beginning of the STAR program, which allows for ample observation of pre and post treatment outcomes, tests of critical model assumptions, and for dynamic effects of the program.

D. Variables

1. *Measured variables*

Our outcome of interest is semi-logged precinct-month counts of STAR-related types of criminal offenses. The City of Denver codes recorded criminal offenses into fifteen overarching categories, including aggravated assault, arson, auto theft, burglary, drug and alcohol offenses, larceny, murder, public disorder, robbery, sexual assault, theft from motor vehicles, traffic accidents, white collar crimes, other crimes against individuals, and all other crimes. These categories give some sense of the types of crimes that might be related to the STAR programs aims but continue to carry a substantial amount of noise for our treatment estimates. From those 15 categories, offenses are differentiated by 199 different types. We coupled information on offense type descriptions and data on their frequencies with independent rater coding to identify those offenses that are STAR-relevant and those that are not. We measure treatment status by capturing each precinct’s monthly participation in the STAR program. Using these data, we construct a simple binary indicator equal to 1 for precinct-month observations from precincts who participate in STAR during a given month (i.e., a “static” measure of treatment). We also use the timing of STAR participation to define less restrictive and flexibly dynamic measures of program participation. These include binary indicators for the month that the program began (June 2020) and separate indicators for being one through five months after that first participation month. These measures flexibly allow for the initial participation in STAR to have effects that increase or decline over time.

2. *Indices*

From the City of Denver's 15 overarching criminal offense categories, we differentiate those offenses that are most related to the types of calls that the STAR team will respond to from other types of offenses that are unlikely to either substitute for a noncriminal STAR team visit or would result from an escalation of such non-criminal offenses. From those 15 offense categories, offenses are differentiated by 199 different types. We coupled information on offense type descriptions and data on their frequencies with independent rater coding to identify those offenses that are STAR-relevant and those that are not.

E. Analysis Plan

1. *Statistical models*

Our main confirmatory analysis is based on a difference-in-differences (DD) design, which assumes that STAR activity in a given precinct and month leads to a constant, one-time change in STAR-related criminal offenses for participating precincts. We do so by comparing changes in these outcomes among precincts participating in STAR to outcomes of precincts that either never participated or had yet to participate in STAR. The outcome will be a semi-logged count of STAR-related criminal offenses. The predictors will be (1) an indicator of a treated precinct in a treated month, (2) precinct fixed effects, and (3) month fixed effects. Standard errors will be clustered at the precinct level.

2. *Transformations*

We use precinct-month counts of STAR-related criminal offenses in the panel data to estimate the effects of the STAR program on the number of offenses committed in each precinct. We transform the outcome variable, which is the semi-logged count of STAR-related criminal offenses for precinct p in month m.

3. *Inference criteria*

We will make inferences of our confirmatory analysis using two-tailed tests and p-values of $p<.10$. We will report p-values differently based on thresholds of $p<.01$, $p<.05$, and $p<.10$.

4. *Data exclusion*

We exclude data prior to December 2019 and after November 2020.

5. *Missing data*

There are no instances of missing precinct-month data, including criminal offenses recorded in a given precinct-month. Thus, we observe no missing data for our confirmatory analyses. However, in some exploratory analyses we examine program effects at the precinct-week level. For instances in which there are no STAR-related

offenses in a given week, we will replace the missing value with the natural log of 0.5. We do the same for STAR-unrelated offenses in exploratory analyses.

6. *Exploratory analysis*

We will conduct a number of exploratory analyses. First, to test for time-varying treatment effects, we next employ a semi-dynamic DD model that unrestrictively allows for treatment effects unique to the month immediately after a precinct first participates and up to seven months later. We then test the equivalence of these coefficients of interest using the null hypothesis of a constant treatment effect. Second, we will conduct an "event study" analysis. A crucial maintained assumption of our DD approach is that the month-to-month outcome changes among "control" precincts (i.e., those without a change in treatment status) provide a valid counterfactual for what would have changed for treatment precincts in the absence of treatment. This "parallel trends" assumption is fundamentally untestable. However, we can provide qualified evidence on the validity of this important assumption through unrestrictive "event study" specifications that allow us to examine whether treatment and control group precincts had similar month-to-month changes in outcomes prior to the onset of treatment. To the extent that this hypothesis is true, it is consistent with the parallel trends assumption. We examine this question through event-study specifications. Third, because these data also include counts of criminal offenses that are unrelated to the STAR programs goals, there is an opportunity to test a "triple diff" (DDD) research design that allows us to account for unobserved disturbances in precinct-month observations. Stacking our data at the precinct-month-(STAR & non-STAR) offense level, the DDD specification includes fixed effects for all two-way interactions. Fourth, we will rerun the static DD model for each of the 15 criminal offense category outcomes reported in the original dataset. Fifth, to test for potential differential effects of the COVID pandemic on criminal offenses, we rerun the confirmatory analysis but only include offenses from March 2020 through November 2020. Sixth, we rerun the confirmatory analysis using a count outcome in a negative binomial precinct fixed effects model. Seventh, we analyze the confirmatory outcome during STAR-eligible and STAR-ineligible times. Eighth, another model tests for spillover effects of the STAR program in precincts adjacent to the participating precincts. Ninth, we examine static and semi-dynamic program effects at the precinct-week level, for STAR-related and STAR-unrelated criminal offenses.

Deviations from Pre-Registration Plan

Our main results do not deviate from the pre-registration plan. However, we have added several additional exploratory analyses not reported in the original pre-registration plan. First, in a robustness check we recode "simple assaults", "simple assaults on police officers", and "disarming a piece officer" as STAR-unrelated offenses (see Table S6). Second, we include a static DD model in which we recode May 2020 as a "treatment" month among STAR-active precincts, to test for anticipation to the program's start (see Table S6). Third, in Table S5 we include an additional pre-trends F-test for only months during COVID restrictions (i.e., March 2020 – May 2020). Fourth, we conduct placebo static DD and event study tests in years prior to our study window (see Table S6 and Figures S5-S7). Finally, we conduct additional robustness checks using a generalized synthetic control (GSC) design and a comparative interrupted time series (CITS) approach (see Table S7).

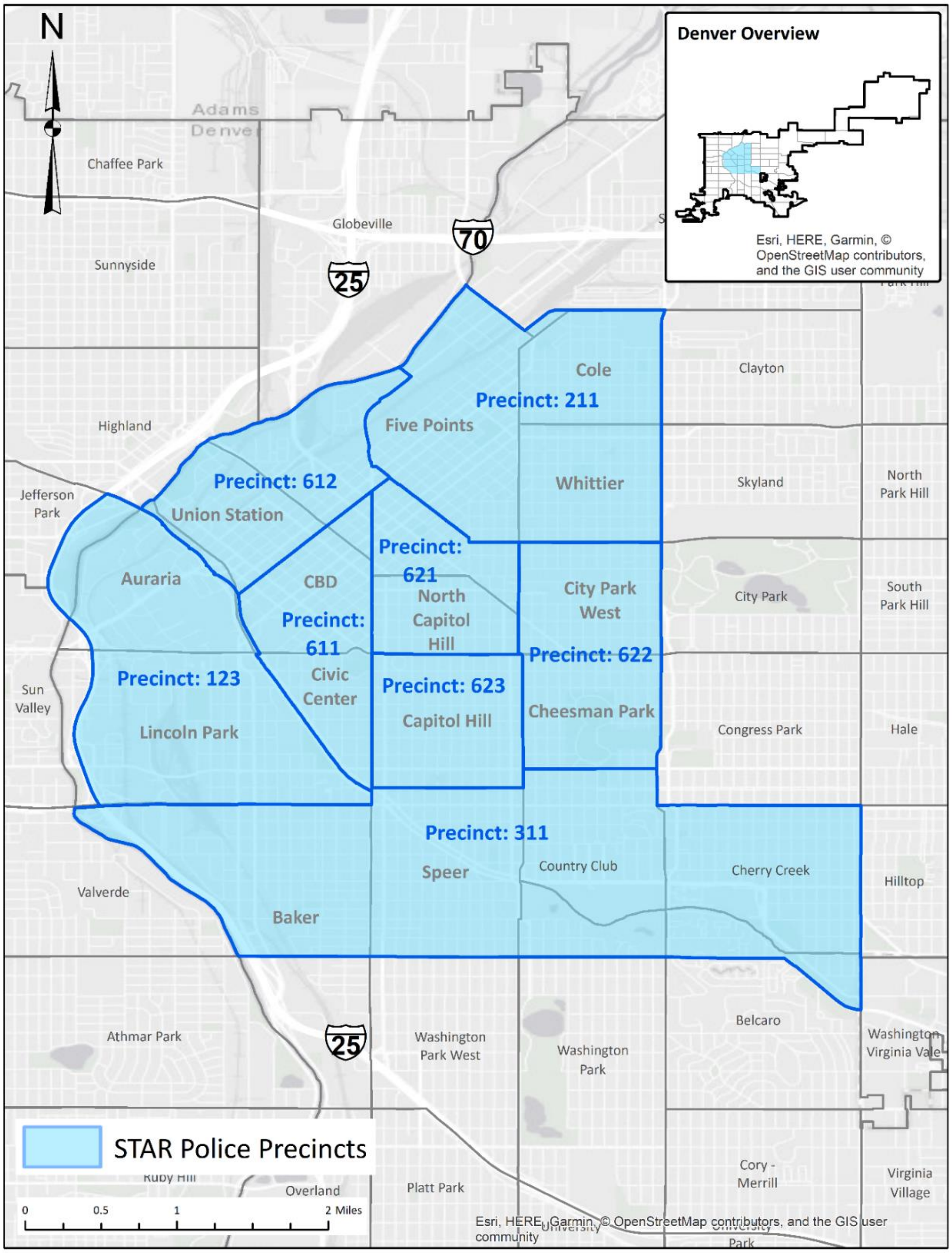

Figure S1. STAR police precincts and neighborhoods. Significantly affected police precincts and neighborhoods are bolded.

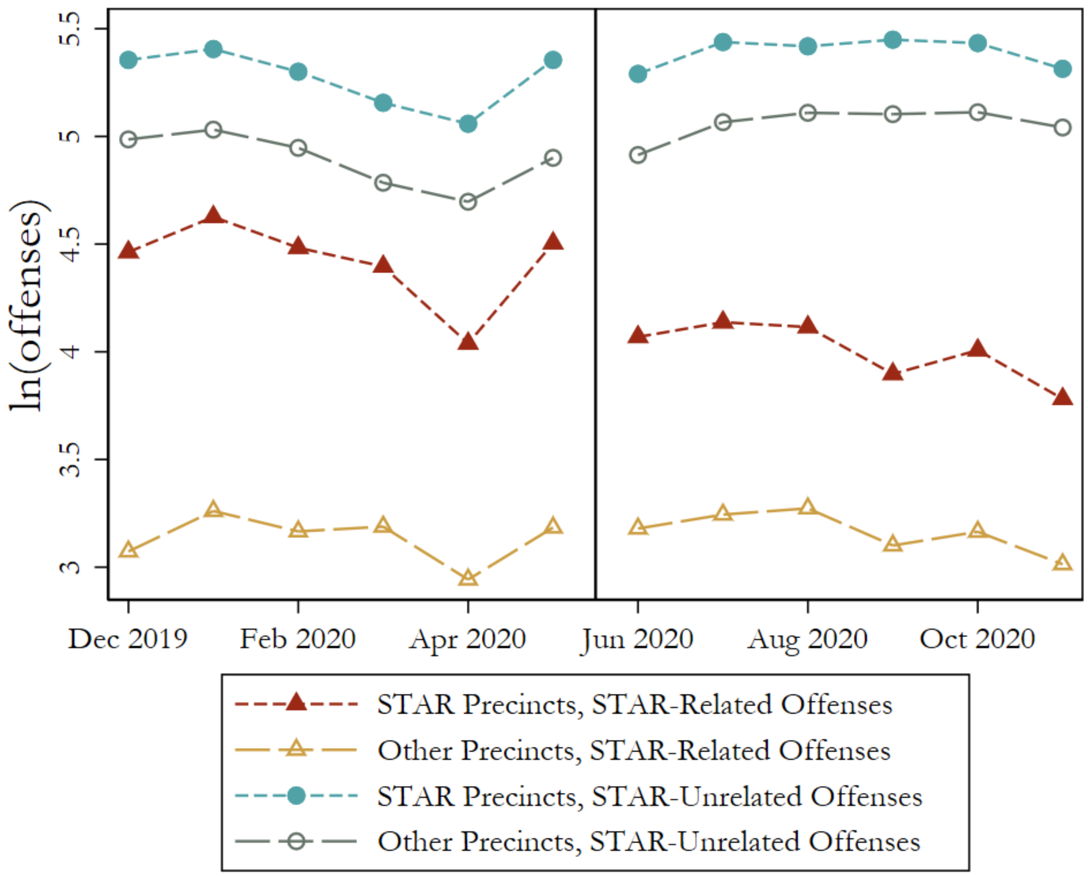

Figure S2. Descriptive Monthly Trends in Offense Types. Conditional means of offenses are based on 432 precinct-month observations of all Denver police precincts, from December 2019 through November 2020. The outcome variables are the natural log of the offense counts, differentiated by STAR and non-STAR precincts as well as by STAR-related and unrelated offenses. The vertical line separates months before and after the STAR pilot program began.

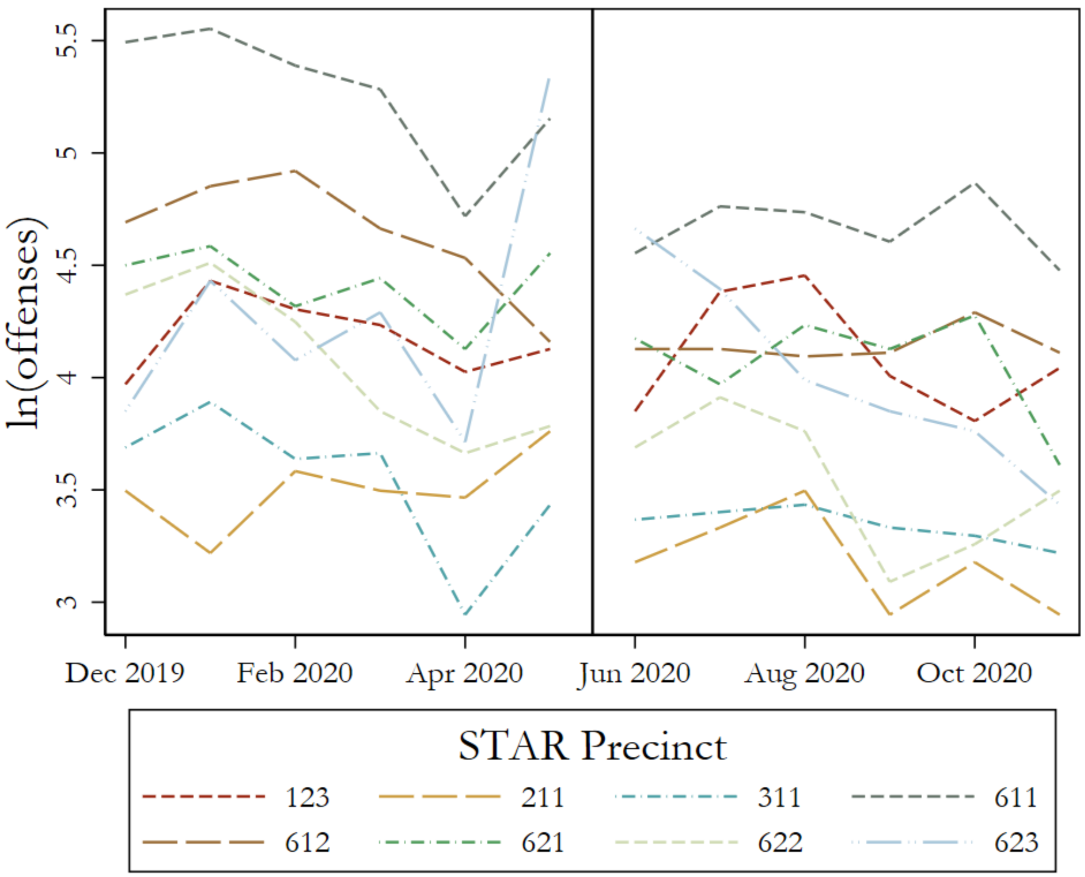

Figure S3. Descriptive Monthly Trends in STAR-Related Offenses among STAR Precincts. Lines are conditional means of STAR-related offenses based on 88 precinct-month observations from December 2019 through November 2020—one for each of the eight STAR-active precincts. The outcome variables are the natural log of the STAR-relevant offense counts. The vertical line separates months before and after the STAR pilot program began.

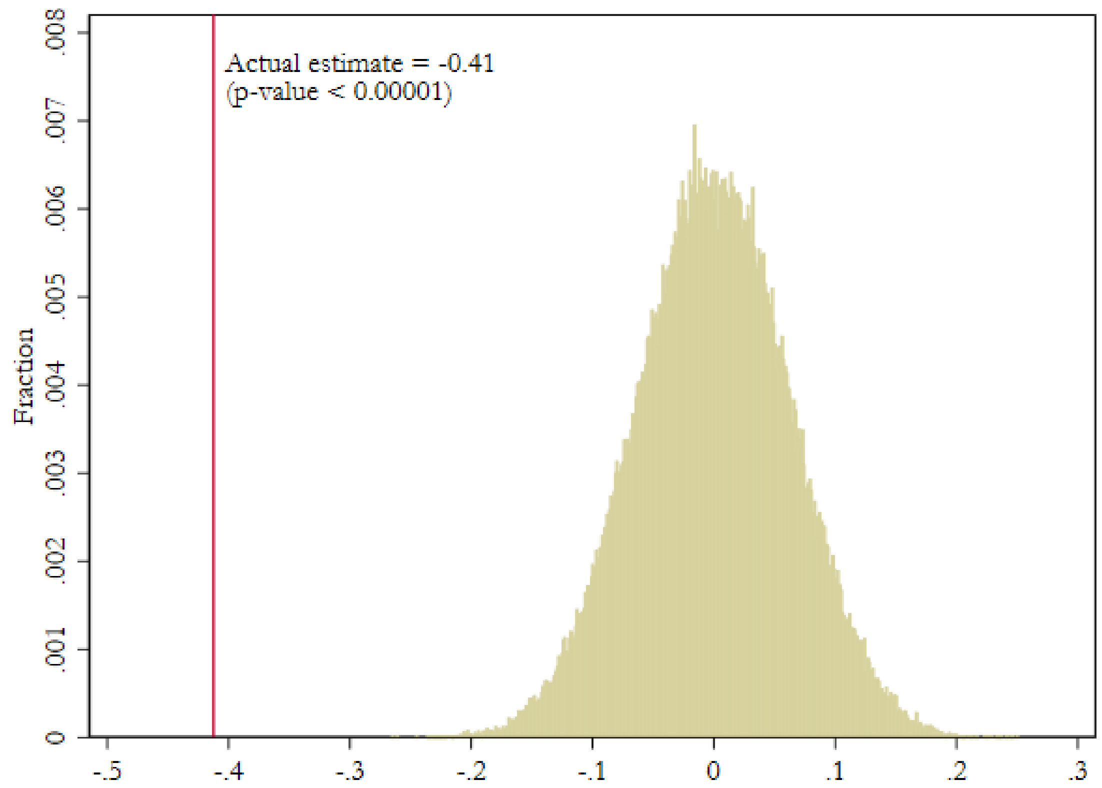

Figure S4. Null Distribution of Estimated Effects (100,000 Replications). Distribution represents results from 100,000 randomized simulations of the data under the assumption that the effects observed from the data were generated at random. The red vertical line represents the actual observed effect size from the data.

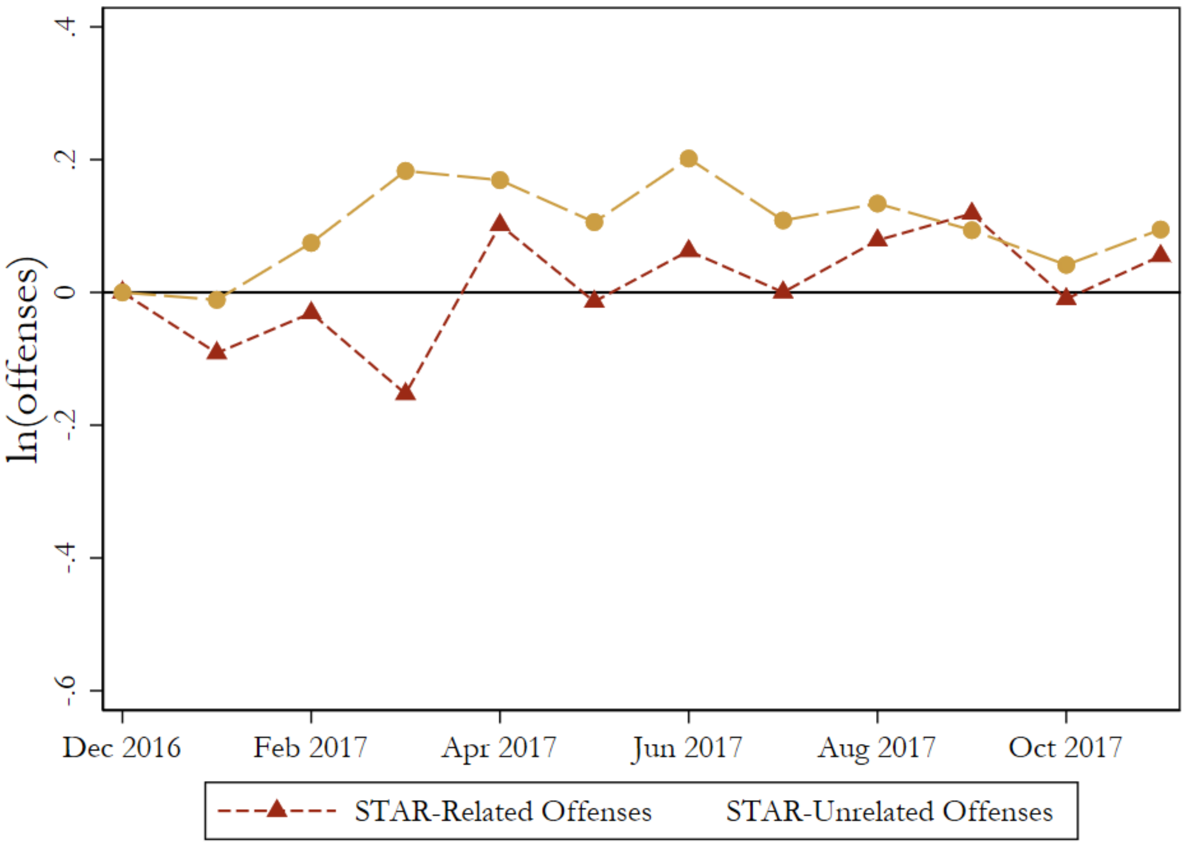

Figure S5. Placebo Event Study (Dec. 2016 – Nov. 2017). This placebo check mirrors the analyses represented in Figure 3 and Table S5, but applied to a time period other than our study window. Specifically, the difference-in-differences (DD) estimates are based on 432 precinct-month observations from December 2016 through November 2017 and condition on precinct fixed effects and month fixed effects. The outcome variables are the natural log of offense counts, differentiated by those that are STAR-related and those that are not. The horizontal line at zero denotes the baseline levels of offenses.

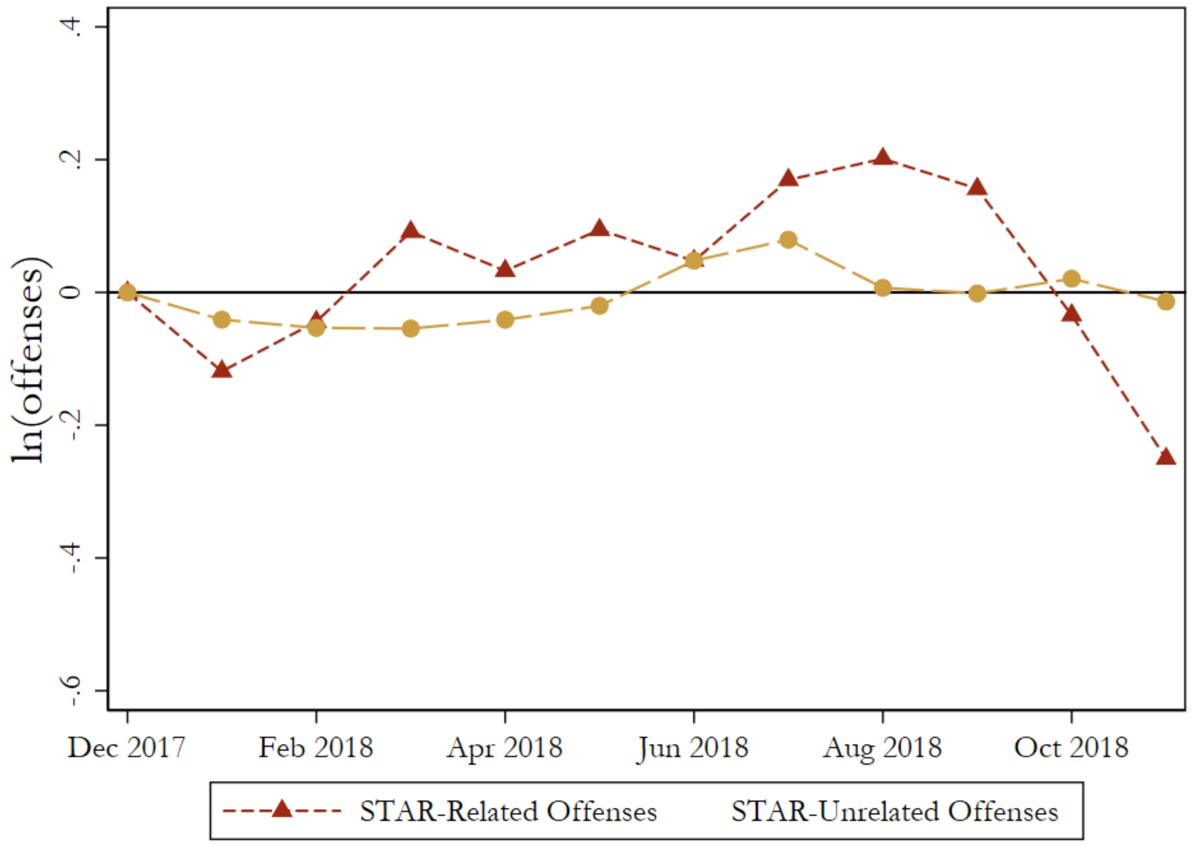

Figure S6. Placebo Event Study (Dec. 2017 – Nov. 2018). This placebo check mirrors the analyses represented in Figure 3 and Table S5, but applied to a time period other than our study window. Specifically, the difference-in-differences (DD) estimates are based on 432 precinct-month observations from December 2017 through November 2018 and condition on precinct fixed effects and month fixed effects. The outcome variables are the natural log of offense counts, differentiated by those that are STAR-related and those that are not. The horizontal line at zero denotes the baseline levels of offenses.

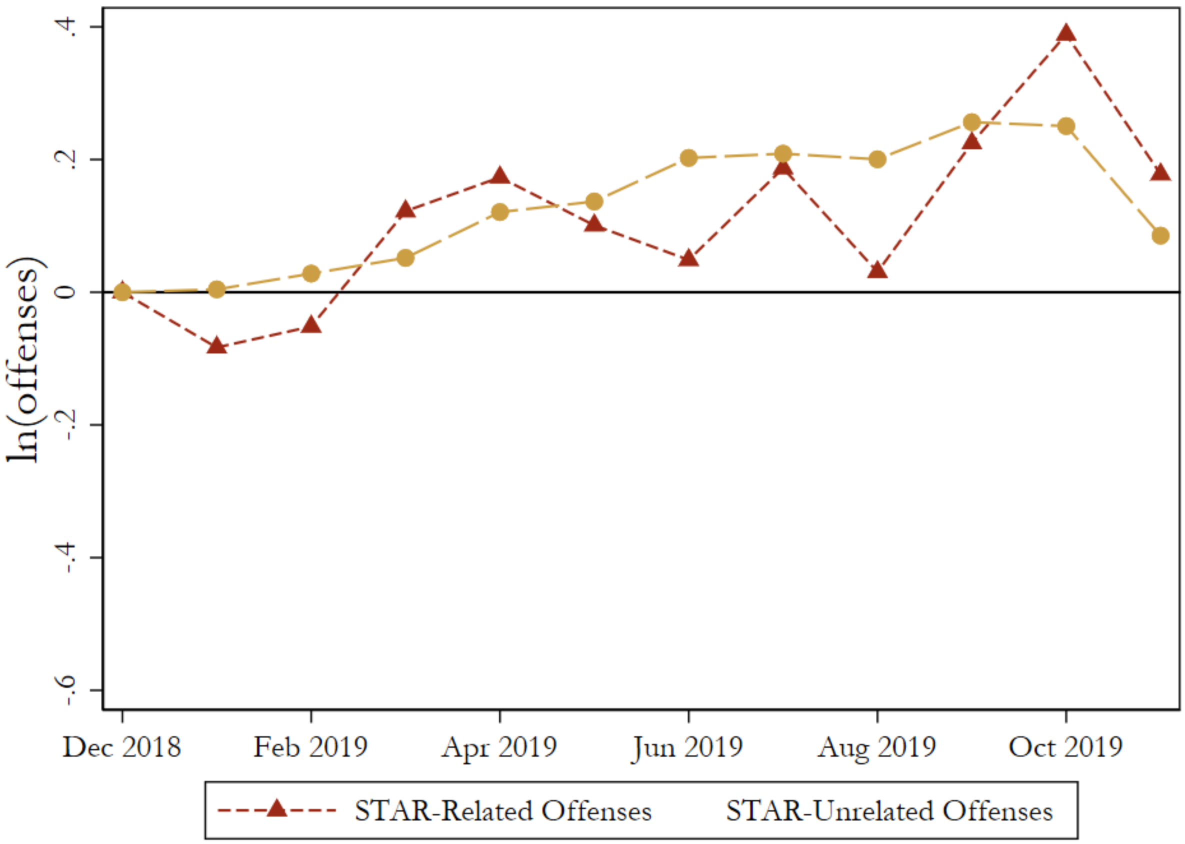

Figure S7. Placebo Event Study (Dec. 2018 – Nov. 2019). This placebo check mirrors the analyses represented in Figure 3 and Table S5, but applied to a time period other than our study window. Specifically, the difference-in-differences (DD) estimates are based on 432 precinct-month observations from December 2018 through November 2019 and condition on precinct fixed effects and month fixed effects. The outcome variables are the natural log of offense counts, differentiated by those that are STAR-related and those that are not. The horizontal line at zero denotes the baseline levels of offenses.

Neighborhood	Population	% College Degree	Median HH Income	% People of Color All	Black	Latinx	Asian	Other
Auraria	778	8%	$ 86,875	30%	1%	23%	0%	6%
Baker	6,568	53%	$ 75,973	35%	4%	24%	2%	5%
Capitol Hill	9,309	64%	$ 63,532	14%	2%	6%	2%	4%
CBD	6,916	27%	$ 38,888	62%	13%	44%	2%	3%
Cheesman Park	5,339	51%	$ 60,514	32%	11%	14%	3%	4%
City Park West	4,552	26%	$ 63,250	63%	14%	47%	0%	2%
Civic Center	18,924	46%	$ 70,971	39%	15%	16%	2%	7%
Cole	12,061	63%	$ 66,120	18%	2%	10%	4%	3%
Five Points	3,032	67%	$ 197,813	8%	1%	3%	0%	3%
Lincoln Park	16,304	60%	$ 54,762	23%	4%	11%	4%	4%
North Capitol Hill	6,754	60%	$ 69,668	26%	6%	16%	2%	2%
Speer	2,256	59%	$ 105,962	25%	9%	10%	5%	2%
Union Station	4,491	47%	$ 75,323	29%	3%	16%	4%	7%
Whittier	7,500	71%	$ 95,487	17%	1%	5%	9%	2%
All STAR	104,784	48%	$ 72,870	33%	8%	19%	3%	4%
All Non-STAR	573,683	36%	$ 77,596	44%	8%	30%	3%	3%
City-Wide	678,467	46%	$ 59,179	46%	10%	33%	4%	3%

Table S1. STAR Pilot Program Neighborhood Demographics. Data come from 2019 American Community Survey data, retrieved from https://www.denvergov.org/opendata/dataset/american-community-survey-nbrhd-2015-2019. All the listed neighborhoods except Whittier are characterized as “displacement-vulnerable.”

STAR-Related Offenses				
Simple assault on police	Possession of cocaine	Possession of synthetic narcotic	Loitering	Property crimes - other
Simple Assault	Possession of hallucinogenic drug	False imprisonment	Obstructing government operation	Public fighting
Criminal mischief - other	Possession of heroin	Harassment	Failure to obey police order	Public order offense - other
Criminal trespassing	Possession of marijuana	Harassment of a sexual nature	Giving police false information	Public peace - other
Disarming a peace officer	Possession of methamphetamine	Indecent exposure	Police interference	Reckless endangerment
Disturbing the peace	Possession of opium or derivative	Liquor law violation - other	Obstructing criminal investigation	Threatening to injure
Possession of a barbiturate	Possession of drug paraphernalia	Possession of liquor	Resisting arrest	
STAR-Unrelated Offenses				
Accessory/conspiracy to crime	Selling cocaine	Fraud by check due to insufficient funds	Inciting a riot	Pocket picking
	Forgery to obtain drugs	Gambling - betting or wagering	Robbery of a bank	Purse snatching without force
Aggravated assault	Fraud to obtain drugs	Running a gambling operation	Robbery of a business	Shoplifting
Aggravated assault - domestic violence	Manufacture of hallucinogenic drug	Gambling - gaming operation	Carjacking - armed	Theft of construction equipment
Altering VIN number	Selling of hallucinogenic drug	Harassment - domestic violence	Forcible purse snatching	Theft of trailer
Possession of dangerous animal	Selling of heroine	Stalking - domestic violence	Robbery of a person in a residence	Unauthorized use of credit/debit card
Arson of a business	Manufacture/sell other dangerous drugs	Conspiracy to commit homicide	Robbery of a person in the open	Habitual traffic offender
Arson - other	Cultivation of marijuana	Homicide by a family member	Unlawful sexual contact	Impound abandoned vehicle
Arson to a public building	Selling of marijuana	Homicide by negligence	Rape	Traffic - other
Arson of a residence	Selling of methamphetamine	Homicide by other means	Rape by person in position of trust	Vehicular assault
Arson of a vehicle	Manufacture of methamphetamine	Homicide of a police officer with a gun	Sexual assault with an object	Vehicular homicide
Aggravated assault to police using gun	Selling of opium or an opium derivative	Illegal dumping	Sexual assault - position of trust	Traffic accident
Assault - domestic violence	Other dangerous drugs - PCS	Impersonation of a police officer	Sexual assault - fondling adult	Traffic accident - DUI DUID
Bomb threat	Selling a synthetic narcotic drug	Intimidation of a witness	Sexual assault - non rape	Traffic accident - hit and run
Bribery	Eavesdropping	Kidnap an adult	Sodomy of adult using force	Vehicular eluding
Burglary/auto theft of a business	Escape of a prisoner	Domestic violence kidnapping	Failure to register as sex offender	Vehicular eluding - no chase
Burglary and auto theft at a business - forced entry	Aiding the escape of a prisoner	Manufacture of liquor	Sex offender registration violation	Violation of court order
Burglary and auto theft at residence	Possession of an explosive device	Liquor - misrepresenting age	Buy, sell, receive stolen property	Violation of custody order
Burglary and auto theft at a residence - forced entry	Use of an explosive device	Illegal sale of liquor	Theft of bicycle	Violation of restraining order
Burglary of a business - forced entry	Possession of explosive device	Littering	Theft by confidence game	Altering weapon serial number
Burglary of a business	Extortion	Threatening to injure with weapon	Embezzlement by an employee	Possession of weapon
Possession of burglary tools	Failure to report abuse	Money laundering	Failure to return rental vehicle	Carrying concealed weapon
Burglary of a residence - forced entry	Possession of fireworks	Manufacture of obscene material	Theft from a building	Carrying prohibited weapon
Burglary of a residence	Forgery of checks	Possession of obscene material	Theft from a mailbox	Weapon fired into occupied building
Burglary of a safe	Counterfeiting an object	Other environmental or animal offense	Theft from a yard	Weapon fired into occupied vehicle
Burglary of a vending machine	Forgery - other	Parole violation	Theft of fuel by driving off	Flourishing a weapon
Smuggle contraband to a prisoner	Possession of forged credit/debit card	Pawn broker violation	Theft of items from vehicle	Weapon violation - other
Possession of contraband	Possession of a forged instrument	making a false report to police	Theft of cable services	Possession of illegal weapon
Criminal mischief - graffiti	Possession of a counterfeiting device	Probation violation	Theft of motor vehicle	Unlawful discharge of weapon
Criminal mischief to a motor vehicle	Fraud by telephone	Aiding the act of prostitution	Theft of rental property	Unlawful sale of weapon
Curfew violation	Fraud by use of computer	Engaging in prostitution	Theft of services	Window peeping
Manufacture of a barbiturate	Criminal impersonation	Pimping for prostitution	Theft - other	Wiretapping
Selling a barbiturate	Identity theft	Engaging in a riot	Theft of parts from a vehicle	Cruelty to animals

Table S2. Offense Type Codes Differentiated by STAR-Related and STAR-Unrelated offenses. Offenses were differentiated using two independent coders who interpreted STAR guidelines for dispatching clinicians to mental-health and substance-abuse calls.

Variable	Mean	SD	Minimum	Maximum	Percent STAR-Related Offenses
Active STAR Program	0.11	0.31	0	1	25.1
STAR-related offenses	33.7	30.9	5	258	100
STAR-unrelated offenses	159.6	54.6	27	406	0.0
Total offenses	193.3	75.3	48	606	21.1
Offenses by category					
Alcohol and drugs	6.3	7.6	0	64	69.8
Aggravated Assault	6.8	4.9	0	26	0.0
Auto theft	18.7	10.5	1	66	0.0
Burglary	11.8	6.8	0	42	0.0
Disorderly conduct	22.6	14.6	2	168	54.8
Larceny	22.7	15.2	1	87	0.0
Theft from motor vehicle	23.2	12.6	1	88	0.0
Traffic offenses	35.7	19.0	1	119	0.0
Other crimes against people	9.3	6.7	0	43	71.6
All other offenses	28.8	24.9	1	204	35.5

Table S3. Descriptive Statistics. The sample is based on Denver's 36 police precincts observed in each of 12 months from December 2019 through November 2020 (n = 432 precinct-month observations). Due to the very low instances of arson (0.2%), murder (0.1%), robbery (2.8%), sexual assault (1.5%), and white-collar offenses (2.7%), we do not include those STAR-unrelated offense categories in the table.

	DD					
	STAR-Related Offenses		STAR-Unrelated Offenses		DDD	
Independent Variables	(1)	(2)	(3)	(4)	(5)	(6)
Active STAR program	-0.41***		-0.05		-0.36***	
	(0.07)		(0.04)		(0.05)	
Adoption month: June 2020		-0.34***		0.01		-0.34***
		(0.12)		(0.06)		(0.09)
1-month lag: July 2020		-0.36***		-0.02		-0.33***
		(0.11)		(0.06)		(0.10)
2-month lag: August 2020		-0.39***		-0.06*		-0.32***
		(0.10)		(0.04)		(0.09)
3-month lag: September 2020		-0.47***		-0.04		-0.43***
		(0.11)		(0.05)		(0.10)
4-month lag: October 2020		-0.45***		-0.07		-0.38***
		(0.08)		(0.06)		(0.10)
5-month lag: November 2020		-0.48***		-0.10		-0.38***
		(0.11)		(0.06)		(0.10)
p value (H_0: $\delta_0=\delta_{-1}=\delta_{-2}=\delta_{-3}=\delta_{-4}=\delta_{-5}$)		0.91		0.68		0.90

Table S4. Estimated Effects of the STAR Program on Criminal Incidents. The difference-in-differences (DD) estimates are based on 432 precinct-month observations and condition on precinct fixed effects and month fixed effects. The difference-in-difference-in- differences (DDD) estimates are based on the stacked precinct-month data for STAR and non-STAR offenses (n = 864). The DDD estimates condition on fixed effects unique to each category of the following 2-way interactions: precinct-by-month, precinct-by-STAR offense, and month-by-STAR offense. The outcome variables are the natural log of the offense counts. Standard errors, clustered at the precinct level, are in parentheses. *** p<0.01, ** p<0.05, * p<0.10.

	Offense Type	
	STAR-Related	STAR-Unrelated
Independent Variables	(1)	(2)
5-month lead	0.02	0.03
	(0.12)	(0.05)
4-month lead	-0.02	0.01
	(0.10)	(0.05)
3-month lead	-0.11	0.03
	(0.12)	(0.07)
2-month lead	-0.15	0.03
	(0.13)	(0.12)
1-month lead	-0.07	0.12
	(0.24)	(0.13)
Precinct participated in June 2020	-0.39**	0.04
	(0.19)	(0.10)
Precinct participated in July 2020	-0.41**	0.01
	(0.17)	(0.09)
Precinct participated in August 2020	-0.44***	-0.03
	(0.16)	(0.07)
Precinct participated in September 2020	-0.52***	-0.00
	(0.17)	(0.08)
Precinct participated in October 2020	-0.50***	-0.04
	(0.13)	(0.09)
Precinct participated in November 2020	-0.53***	-0.06
	(0.14)	(0.08)
p value (H_0: $\delta_5=\delta_4=\delta_3=\delta_2=\delta_1=0$)	0.71	0.87
p value (H_0: $\delta_3=\delta_2=\delta_1=0$)	0.60	0.62
p value (H_0: $\delta_0=\delta_{-1}=\delta_{-2}=\delta_{-3}=\delta_{-4}=\delta_{-5}$)	0.91	0.69

Table S5. Event-study estimates. The difference-in-differences (DD) estimates are based on 432 precinct-month observations and condition on precinct fixed effects and month fixed effects. The outcome variables are the natural log of the STAR-related offense counts. Standard errors, clustered at the precinct level, are in parentheses. *** p<0.01, ** p<0.05, * p<0.10.

Model	DD Estimate
Clustering corrected for finite sample bias	-0.41***
	(0.07)
Poisson fixed effects model	-0.45***
	(0.03)
Negative binomial fixed effects model	-0.42***
	(0.06)
Without Precinct 311 (n=420)	-0.43***
	(0.07)
Post-pandemic months (March-Nov 2020) only (n=324)	-0.38***
	(0.06)
Effects during STAR-eligible days and times	-0.37***
	(0.13)
Effects during STAR-ineligible days and times	-0.44***
	(0.06)
Recoding simple assaults as STAR-unrelated offenses	-0.49***
	(0.08)
Recoding May 2020 as a treatment month	-0.36***
	(0.09)
Effects in STAR-adjacent precincts (all times)	0.02
	(0.08)
Effects in STAR-adjacent precincts (eligible times)	-0.04
	(0.14)
Effects in STAR-adjacent precincts (ineligible times)	0.05
	(0.08)
Placebo effects, June - Nov. 2017 (Dec. 2016 start)	0.08
	(0.08)
Placebo effects, June - Nov. 2018 (Dec. 2017 start)	0.04
	(0.09)
Placebo effects, June - Nov. 2019 (Dec. 2018 start)	0.13*
	(0.05)

Table S6. Alternative specifications. The difference-in-differences (DD) estimates are based on 432 precinct-month observations (unless otherwise noted). All models condition on month fixed effects. All models condition on precinct fixed effects. The outcome variables are the natural log of the STAR-relevant offense counts. Standard errors, clustered at the precinct level, are in parentheses. *** p<0.01, ** p<0.05, * p<0.10.

	GSC		CITS	
	STAR Offenses	Non-STAR Offenses	STAR Offenses	Non-STAR Offenses
Treatment X Post	-0.46***	0.02	-0.24**	-0.03
	(0.14)	(0.11)	(0.10)	(0.05)
Treatment X Post X Trend	--	--	0.00	-0.04
			(0.06)	(0.03)
Treatment X Trend	--	--	-0.03	0.02
			(0.04)	(0.03)

Table S7. Comparative interrupted time series estimates. The dependent variable is the natural log of the stated offenses (n = 432 precinct-month observations). The first two columns report estimates based on generalized synthetic control (GSC; *45*) and bootstrapped standard errors (1,000 replications). The next two columns report estimates based on a comparative interrupted time-series (CITS) specification (*46*) and standard errors clustered at the precinct level. The CITS specifications also condition on precinct fixed effects and month fixed effects. *** $p<0.01$, ** $p<0.05$, * $p<0.10$.

Outcome Variable	DD Estimate
All offenses	-0.15***
	(0.05)
Alcohol and drugs	-0.53**
	(0.20)
Aggravated Assault	0.05
	(0.15)
Auto theft	0.15*
	(0.09)
Burglary	-0.06
	(0.09)
Disorderly conduct	-0.20***
	(0.06)
Larceny	-0.06
	(0.08)
Theft from motor vehicle	0.04
	(0.09)
Traffic offenses	-0.04
	(0.06)
Other crimes against people	-0.14*
	(0.07)
All other offenses	-0.13
	(0.12)

Table S8. Estimated Effects by Offense Category. The difference-in-differences (DD) estimates are based on 432 precinct-month observations and condition on precinct fixed effects and month fixed effects. Due to the very low instances of arson, murder, robbery, sexual assault, and white-collar offenses, we do not include those STAR-unrelated offense categories. The outcome variables are the natural log of the offense counts. Standard errors, clustered at the precinct level, are in parentheses. *** $p<0.01$, ** $p<0.05$, * $p<0.10$.

REFERENCES AND NOTES

1. C. Lum, D. S. Nagin, Reinventing American policing. *Crime Justice* **46**, 339–393 (2017).

2. C. F. Manski, D. S. Nagin, Assessing benefits, costs, and disparate racial impacts of confrontational proactive policing. *Proc. Natl. Acad. Sci.* **114**, 9308–9313 (2017).

3. S. N. Durlauf, Assessing racial profiling. *Econ. J.* **116**, F402-F426 (2006).

4. National Academies of Sciences, Medicine and Engineering, *Proactive Policing: Effects on Crime and Communities* (National Academies Press, 2018).

5. A. Irwin, B. Pearl, The community responder model: How cities can send the right responder to every 911 call (Center for American Progress, 2020).

6. S. R. Neusteter, M. O'Toole, M. Khogali, A. Rad, F. Wunschel, S. Scaffidi, M. Sinkewicz, M. Mapolski, P. De Grandis, D. Bodah, H. Pineda, Understanding police enforcement: A multicity 911 analysis (Vera Institute of Justice, 2020).

7. C. Lum, C. S. Koper, X. Wu, Can we really defund the police? A nine-agency study of police response to calls for service. *Police Q.* **2021**, 1–26 (2021).

8. M. A. Cohen, A. R. Piquero, New evidence on the monetary value of saving a high risk youth. *J. Quant. Criminol.* **25**, 25–49 (2009).

9. M. Jagannathan, "As activists call to defund the police, mental-health advocates say 'the time is now' to rethink public safety," *MarketWatch*, 19 June 2020.

10. M. G. McDowell, L. A. Fernandez, 'Disband, disempower, and disarm': Amplifying the theory and practice of police abolition. *Critical Criminol.* **26**, 373–391 (2018).

11. US Department of Health & Human Services, Mental health myths and facts (2017); www.mentalhealth.gov [accessed 8 January 2022].

12. M. E. Rueve, R. S. Welton, Violence and mental illness. *Psychiatry* **5**, 34–48 (2008).

13. “Responding to persons experiencing a mental health crisis” (IACP Law Enforcement Policy Center, 2018).

14. D. A. Fuller, H. R. Lamb, M. Biasotti, J. Snook, Overlooked in the undercounted: The role of mental illness in fatal law enforcement encounters (Treatment Advocacy Center, 2015).

15. B. Dahlberg, “Rochester Hospital released Daniel Prude hours before fatal encounter with police,” *National Public Radio*, 29 September 2020.

16. T. Elfrink, “He’s a small child’: Utah police shot a 13-year-old boy with autism after his mother called 911 for help,” *The Washington Post*, 8 September 2020.

17. “Walter Wallace Jr. struggled with mental health issues, family says,” *NBC 10 Philadelphia*, 29 October 2020.

18. C. Lum, Perspectives on policing: Cynthia Lum. *Annu. Rev. Criminol.* **4**, 19–25 (2021).

19. M. T. Compton, M. Bahora, A. C. Watson, J. R. Oliva, A comprehensive review of extant research on Crisis Intervention Team (CIT) programs. *J.Am. Acad. Psychiatry Law* **36**, 47–55 (2008).

20. G. K. Shapiro, A. Cusi, M. Kirst, P. O’Campo, A. Nakhost, V. Stergiopoulos, Co-responding police-mental health programs: A review. *Adm. Policy Ment. Health* **42**, 606–620 (2015).

21. M. S. Morabito, J. Savage, L. Sneider, K. Wallace, Police response to people with mental illnesses in a major U.S. city: The Boston experience with the co-responder model. *Vict. Offenders* **13**, 1093–1105 (2018).

22. C. White, D. Weisburd, A co-responder model for policing mental health problems at crime hot spots: Findings from a pilot project. *Policing* **12**, 194–209 (2018).

23. S.-M. Yang, C. Gill, L. C. Kanewske, P. S. Thompson, Exploring police response to mental health calls in a nonurban area: A case study of Roanoke County, Virginia. *Vict. Offenders* **13**, 1132–1152 (2018).

24. S. Puntis, D. Perfect, A. Kirubarajan, S. Bolton, F. Davies, A. Hayes, E. Harriss, A. Molodynski, A systematic review of co-responder models of police mental health 'street' triage. *BMC Psychiatry* **18**, 256 (2018).

25. J. Peterson, J. Densley, Is Crisis Intervention Team (CIT) training evidence-based practice? A systematic review. *J. Crime Justice* **41**, 521–534 (2018).

26. S. A. Taheri, Do crisis intervention teams reduce arrests and improve officer safety? A systematic review and meta-analysis. *Crim. Justice Policy Rev.* **27**, 76–96 (2016).

27. C. Seo, B. Kim, N. E. Kruis, Variation across police response models for handling encounters with people with mental illnesses: A systematic review and meta-analysis. *J. Crim. Justice* **72**, 101752 (2020).

28. C. Seo, B. Kim, N. E. Kruis, A meta-analysis of police response models for handling people with mental illnesses: Cross-country evidence on the effectiveness. *Int. Crim. Justice Rev.* **31**, 182–202 (2021).

29. T. Richards, "AOC's defund-the-police pick for NYC mayor will 'embolden the bad guys,' Kerik warns," *The Washington Examiner*, 8 June 2021.

30. R. J. Sampson, S. W. Raudenbush, Systematic social observation of public spaces: A new look at disorder in urban neighborhoods. *Am. J. Sociol.* **105**, 603–651 (1999).

31. T. L. Meares, T. R. Tyler, J. Gardener, Lawful or fair? How cops and laypeople perceive good policing. *J. Crim. Law Criminol.* **105**, 297–344 (2015).

32. G. L. Kelling, J. Q. Wilson, Broken windows. *Atl. Mon.* **249**, 29–38 (1982).

33. C. S. Koper, E. Mayo-Wilson, Police strategies to reduce illegal possession and carrying of firearms: Effects on gun crime. *Campbell Syst. Rev.* **8**, 1–53 (2012).

34. "Senator Wyden to visit CAHOOTS, citing as a model for reform" (White Bird Clinic, 2020).

35. "Gentrification study: Mitigating involuntary displacement" (City and County of Denver, 2016).

36. C. Herring, D. Yarbrough, L. Marie Alatorre, Pervasive penality: How the criminalization of poverty perpetuates homelessness. *Soc. Probl.* **67**, 131–149 (2020).

37. T. Gowan, *Hobos, Hustlers, and Backsliders: Homeless in San Francisco* (Univ. of Minnesota Press, 2010).

38. T. Gowan, The Nexus: Homelessness and incarceration in two American cities. *Ethnography* **3**, 500–534 (2002).

39. D. Mitchell, *The Right to the City: Social Justice and the Fight for Public Space* (Guilford Press, 2003).

40. "DJP helps launch alternative public health emergency response pilot in Denver", press release (Denver Justice Project, 2020).

41. "STAR program evaluation," (Denverite, 8 January, 2021).

42. N. W. Link, J. T. Ward, R. Stansfield, Consequences of mental and physical health for reentry and recidivism: Toward a health-based model of desistance. *Crim.* **57**, 544–573 (2019).

43. D. T. O'Brien, C. Farrell, B. C. Welsh, Broken (windows) theory: A meta-analysis of the evidence for the pathways from neighborhood disorder to resident health outcomes and behaviors. *Soc. Sci. Med.* **228**, 272–292 (2019).

44. J. Hausman, B. H. Hall, Z. Griliches, Econometric models for count data with an application to the patents-R & D relationship. *Econometrica* **52**, 909–938 (1984).

45. Y. Xu, Generalized synthetic control method: Causal inference with interactive fixed effects models. *Polit. Anal.* **25**, 57–76 (2017).

46. W. R. Shadish, T. D. Cook, D. T. Campbell, *Experimental and Quasi-Experimental Designs for Generalized Causal Inference* (Houghton Mifflin, 2002).

47. S. D. Johnson, K. Bowers, A. Hirschfield, New insights into the spatial and temporal distribution of repeat victimization. *Br. J. Criminol.* **37**, 224–241 (1997).

48. J. Peterson, J. Densley, G. Erickson, Evaluation of 'The R-Model' crisis intervention de-escalation training for law enforcement. *Police J.* **93**, 271–289 (2020).

49. J. Hails, R. Borum, Police training and specialized approaches to respond to people with mental illnesses. *Crime Delinq.* **49**, 52–61 (2003).

50. E. Schmelzer, "Call police for a woman who is changing clothes in an alley? A new program in Denver sends mental health professionals instead", *Th*e Denver Post, 6 September 2020.

51. J. E. Pustejovsky, E. Tipton, Small-sample methods for cluster-robust variance estimation and hypothesis testing in fixed effects models. *J. Bus. Econ. Stat.* **36**, 672–683 (2018).
